# Confounder-Matched Deep Learning on Cardiac CT for the Diagnosis of Tetralogy of Fallot: A Proof of Concept

**DOI:** 10.3390/diagnostics16172814

**Published:** 2026-09-01

**Authors:** Elnur Karimov, İnci Zaim Gökbay, Serap Baş

**Affiliations:** 1 Department of Interdisciplinary Developmental Behavioral Disorders and Integrated Approach, Institute of Graduate Studies in Health Sciences, İstanbul University, 34126 İstanbul, Türkiye; 2 Clinic of Pediatric Cardiology, Başakşehir Çam and Sakura City Hospital, University of Health Sciences Turkey, 34480 İstanbul, Türkiye; 3 Department of Artificial Intelligence and Data Engineering, Faculty of Computer and Information Technologies, İstanbul University, 34134 İstanbul, Türkiye; 4 Clinic of Radiology, Başakşehir Çam and Sakura City Hospital, University of Health Sciences Turkey, 34480 İstanbul, Türkiye

**Keywords:** tetralogy of Fallot, cardiac CT, deep learning, transfer learning, confounder matching, explainable AI, Grad-CAM, congenital heart disease

## Abstract

**Background/Objectives:** Tetralogy of Fallot (TOF) is the most common cyanotic congenital heart defect, and cardiac computed tomography (CT) is increasingly central to its anatomical and pre-procedural assessment. Artificial-intelligence research in TOF is dominated by MRI; deep learning on cardiac CT in congenital heart disease exists but addresses multi-class diagnosis and segmentation, and the one binary TOF-versus-control CT study used slice-level validation without confounder control, and, to our knowledge, no CT study reports controlling the confounding intrinsic to a TOF-versus-control comparison. This confounding is structural: TOF is imaged predominantly in infancy, so a naive classifier can learn age, body size, and acquisition protocol rather than pathology. We develop and internally evaluate a confounder-matched, anatomy-guided deep-learning pipeline for TOF on cardiac CT. **Methods:** Contrast-enhanced cardiac CT from a single scanner was de-identified and restricted to one reconstruction (FC15 kernel, 0.5 mm), then matched 1:1 on age and sex, yielding 42 TOF and 42 controls (n = 84); controls were children imaged for suspected but excluded cardiovascular disease, so scan indication, unlike age and sex, was not matched. Standardized volumes were decomposed into four fixed sub-volumes positioned to approximate the components of the diagnostic tetrad: malalignment ventricular septal defect (VSD), overriding aorta, right-ventricular outflow tract (RVOT), and right-ventricular hypertrophy (RVH). Whether each sub-volume contains its named target was audited against independent physician region-of-interest annotations. Per region, a 2.5D transfer-learning classifier (ImageNet ResNet18) and a 3D CNN (DenseNet121) were trained with leak-free patient-level five-fold cross-validation and the branches fused. Optimism was assessed by repeated cross-validation and, for model selection, by nested cross-validation with the component subset and operating point chosen inside an inner loop. Discrimination was reported with bootstrap 95% confidence intervals (CIs); AUROCs were compared by DeLong test, with Benjamini–Hochberg correction applied to a seven-member family (the four within-component comparisons, two hybrid-versus-VSD contrasts, and hybrid versus whole-heart) and other comparisons reported uncorrected. **Results:** Matching removed the age difference (median 0.33 years, IQR 0.17–0.92 vs. 0.33, IQR 0.27–0.73; *p* = 0.86) with balanced sex (*p* = 1.00). The pre-specified four-component hybrid reached AUROC 0.829 (95% CI 0.74–0.91); the VSD region alone reached 0.828 (0.74–0.91), so the tetrad decomposition did not improve accuracy, and the containment audit shows it does not deliver the intended anatomical interpretability either. The 2.5D model exceeded the 3D CNN for every component (0.769–0.828 vs. 0.573–0.656; raw DeLong *p* = 0.007–0.037, Benjamini–Hochberg q up to 0.065 under a seven-member family, the weakest comparison (RVH) not surviving correction). Repeated cross-validation gave 0.811 ± 0.026 and nested cross-validation 0.787 ± 0.029; a stronger backbone with multi-phase data, handcrafted radiomics, and a large CT foundation model did not significantly improve on the matched pipeline. Grad-CAM maps were sensitive to both model weights and labels and superior to a centred-blob null in all eight comparisons and significantly so in seven, but not consistently superior to a resolution-matched random attribution, so no localization claim is made. Calibration was imperfect (slope 0.67) and recalibration gave no net gain; at an in-sample Youden threshold sensitivity was 0.93 and specificity 0.64. Occlusion sensitivity on the whole-heart baseline model showed it relies on the physician-marked septal, aortic and right-ventricular sites 1.8–4.1 times more than distance-matched surrounding tissue, while gross morphometry alone reached 0.651–0.663. Two of the four sub-volumes did not contain their target: the RVOT prior contained the physician annotation in 48.1% of cases and, because the model samples only the central band, excluded it in 99.4%; the RVH box was offset toward the midline, containing the marked target in 43.4% of annotations. Repositioning the priors, leak-free and derived from controls only, did not change discrimination (all *p* ≥ 0.10), and boxes placed at random positions inside the standardized heart reached 0.765 on average against 0.796 for the published priors, a difference this cohort cannot resolve. **Conclusions:** As a proof of concept, confounder-matched deep learning can recognize TOF on cardiac CT. Increasing model capacity did not significantly improve on the matched pipeline; the separate contribution of matching itself was not isolated against an unmatched comparator. Given the small, single-centre sample and the absence of external validation, these findings are hypothesis-generating and require external, multi-centre confirmation before any clinical use.

## 1. Introduction

Tetralogy of Fallot (TOF) is the most common cyanotic congenital heart defect, accounting for 7–10% of congenital cardiac malformations, and it requires lifelong imaging surveillance [1,2]. Its diagnosis rests on a four-component tetrad: a malalignment ventricular septal defect (VSD), an overriding aorta, right-ventricular outflow-tract (RVOT) obstruction, and right-ventricular hypertrophy (RVH), all arising from antero-cephalad deviation of the outlet septum [3]. Echocardiography is the first-line modality, but cross-sectional imaging is increasingly used for anatomical and pre-procedural assessment, and cardiac computed tomography (CT) now complements MRI where MRI is contraindicated, unavailable, or too slow in small children [2,4,5]. As CT becomes more central to the TOF pathway, tools that help standardize and support its interpretation become clinically relevant.

Despite this, artificial-intelligence research in TOF has concentrated largely on cardiac MRI, chiefly segmentation and ventricular quantification [6]. Deep learning on cardiac CT in congenital heart disease is not absent, but it is directed at different questions: the largest published system assigns one of seventeen congenital diagnoses from CT in more than 3750 patients and performs comparably to junior cardiovascular radiologists [7]; the public ImageCHD dataset supports the same multi-class task on 110 annotated volumes [8]; and pulmonary-artery segmentation has recently been used for automated surgical-approach triage in TOF across several centres [9]. None of this work controls the confounding intrinsic to a TOF-versus-control comparison. To our knowledge, no CT study in this literature matches cases and controls on age or sex, or harmonizes acquisition; the studies are compared in Section 4.1. The single prior study to pose TOF detection on CT as a binary classification did so on twenty children represented as eighty individually selected slices, with slice-level rather than patient-level validation and without confounder control or external validation [10]. This is a clinically meaningful gap: the modality gaining ground in practice has not been evaluated under confounder control. Part of this sparsity is clinical rather than technical: echocardiography is the diagnostic reference standard in this condition and the diagnosis is typically established before CT, whose preoperative role is the delineation of pulmonary arterial, aortic, and coronary anatomy rather than the assignment of a diagnostic label [2,11,12], so the unmet need is less an autonomous diagnostic aid than a methodologically sound benchmark. That, in turn, makes TOF-versus-control CT an instructive testbed for the methodological question this study targets: how to obtain an honest internal estimate under confounder control at small scale.

The gap is harder to close than it first appears because of a confounding problem intrinsic to this disease. TOF is diagnosed and repaired predominantly in infancy, whereas control cardiac CTs span all ages; consequently, age, body size, reconstruction kernel, and tube voltage all correlate with disease status. A classifier trained naively on such data can achieve high apparent accuracy by learning the acquisition and age signature rather than the pathology, a failure mode demonstrated for radiographic classifiers [13] and cautioned against in congenital-heart-disease imaging specifically [14]. Any credible CT-based TOF classifier must therefore be designed around this confounding rather than ignoring it. A second obstacle is data scarcity: paediatric cardiac CT cohorts are small, which limits training from scratch and motivates transfer learning, in which a network pretrained on a large dataset is fine-tuned on the domain-specific task [15]. A third is the “black-box” criticism that limits clinical acceptance of deep learning; explainable-AI methods such as Gradient-weighted Class Activation Mapping (Grad-CAM) [16] are widely applied for this purpose, but their faithfulness is contested: no saliency method evaluated in medical imaging satisfies every trustworthiness criterion [17], and agreement with expert localization degrades as the target structure shrinks [18]. Any saliency analysis must therefore be tested against a resolution-matched null rather than accepted on inspection.

In this study, we develop a CT-based, confounder-controlled, anatomy-guided deep-learning pipeline for TOF and evaluate it internally. The contributions are a 1:1 age- and sex-matched, acquisition-harmonized cohort that removes the age difference and equalizes the reconstruction kernel by design rather than by post-hoc adjustment, while leaving a residual tube-voltage imbalance and leaving scan indication and body size unmatched; a decomposition of the standardized heart into four fixed sub-volumes intended as tetrad-component priors, audited against physician annotations that show two of the four do not contain their named target, evaluated with leak-free patient-level cross-validation and with the component subset and decision threshold nested inside an inner loop; and benchmarking of 2.5D transfer learning against 3D CNNs, a whole-heart baseline, radiomics, and a CT foundation model, reported as configuration-specific comparisons with emphasis on confidence intervals and on the limits of a matched-cohort design.

## 2. Materials and Methods

**Figure 1:**
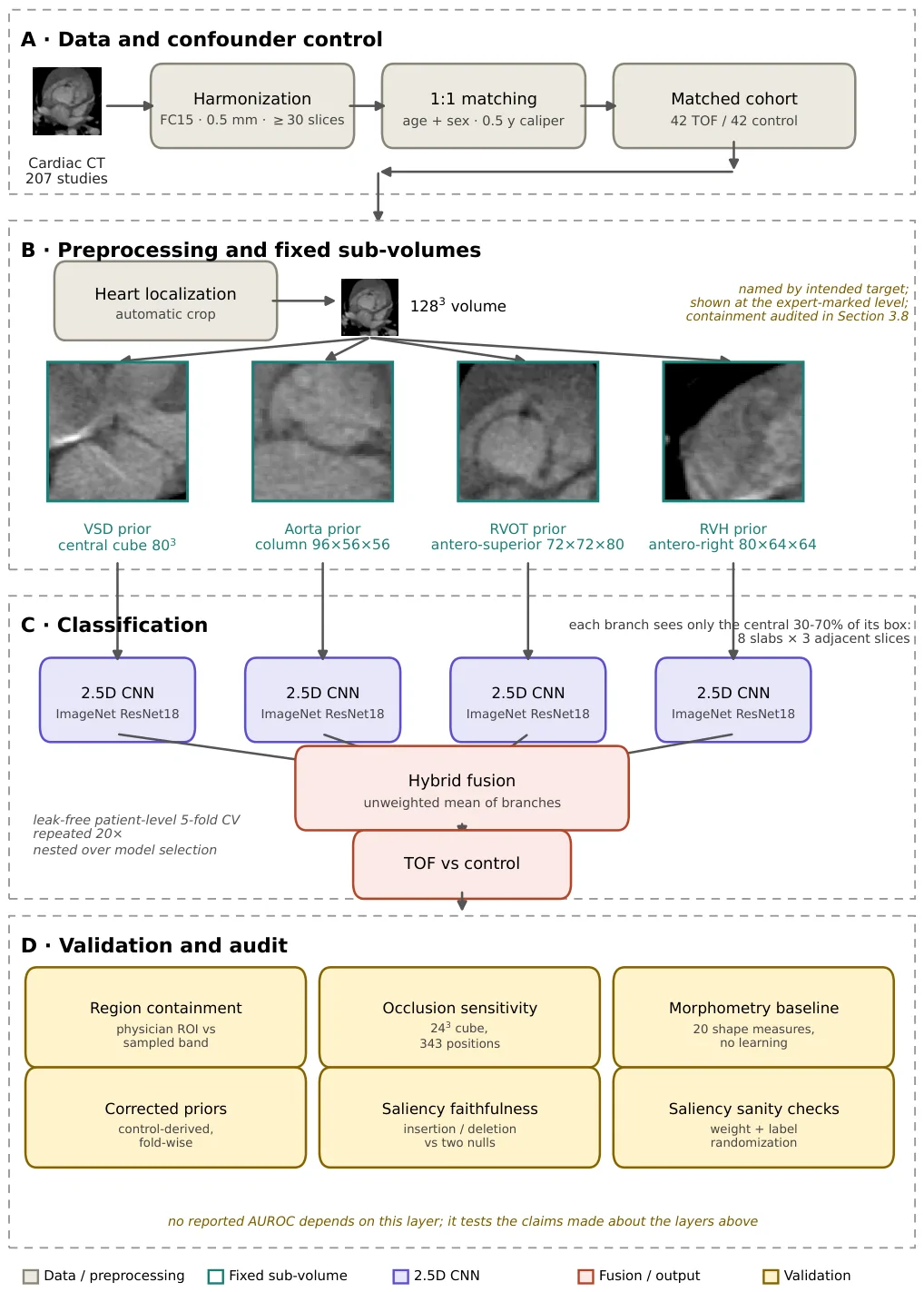
Study flow, analysis pipeline, and validation chain. (**A**) Confounding is removed by design rather than by adjustment: a single reconstruction is retained, then patients are matched 1:1 on age and sex. (**B**) The heart is localized and cropped automatically and every volume is standardized to $128^{3}$, from which four fixed sub-volumes are cut. These are named for the tetrad component each was positioned to capture; whether they contain it was audited separately, and for two of them the answer is no (Section 3.8). (**C**) Each sub-volume feeds a 2.5D transfer-learning branch and the four are averaged. A branch does not see its whole box: slabs are sampled across the central 30–70% of the region depth, which is why a prior can contain a structure and still never present it to the model. (**D**) The validation layer. None of the primary or comparator discrimination estimates depends on it; its purpose is to test the claims made about the layers above, and it is what identified the region-containment failure. Thumbnails are real cardiac CT from the case used in Figure 2, each shown at the axial level of that region’s expert-placed target rather than at the mid-depth of its box, since the two differ by 11 to 28 slices here. The RVH thumbnail accordingly shows mostly extracardiac tissue: that prior is offset from its target, and no level of it depicts right-ventricular hypertrophy.

We followed the CLAIM (Checklist for Artificial Intelligence in Medical Imaging; 42-item) and TRIPOD+AI (27-item) reporting guidelines [19,20,21], with completed item-by-item checklists in Supplementary Tables S7 and S8; the STARD-AI guideline [22] informed the reporting of the diagnostic-accuracy elements, but no completed STARD-AI form is provided. The overall workflow is shown in Figure 1.

**Figure 2:**
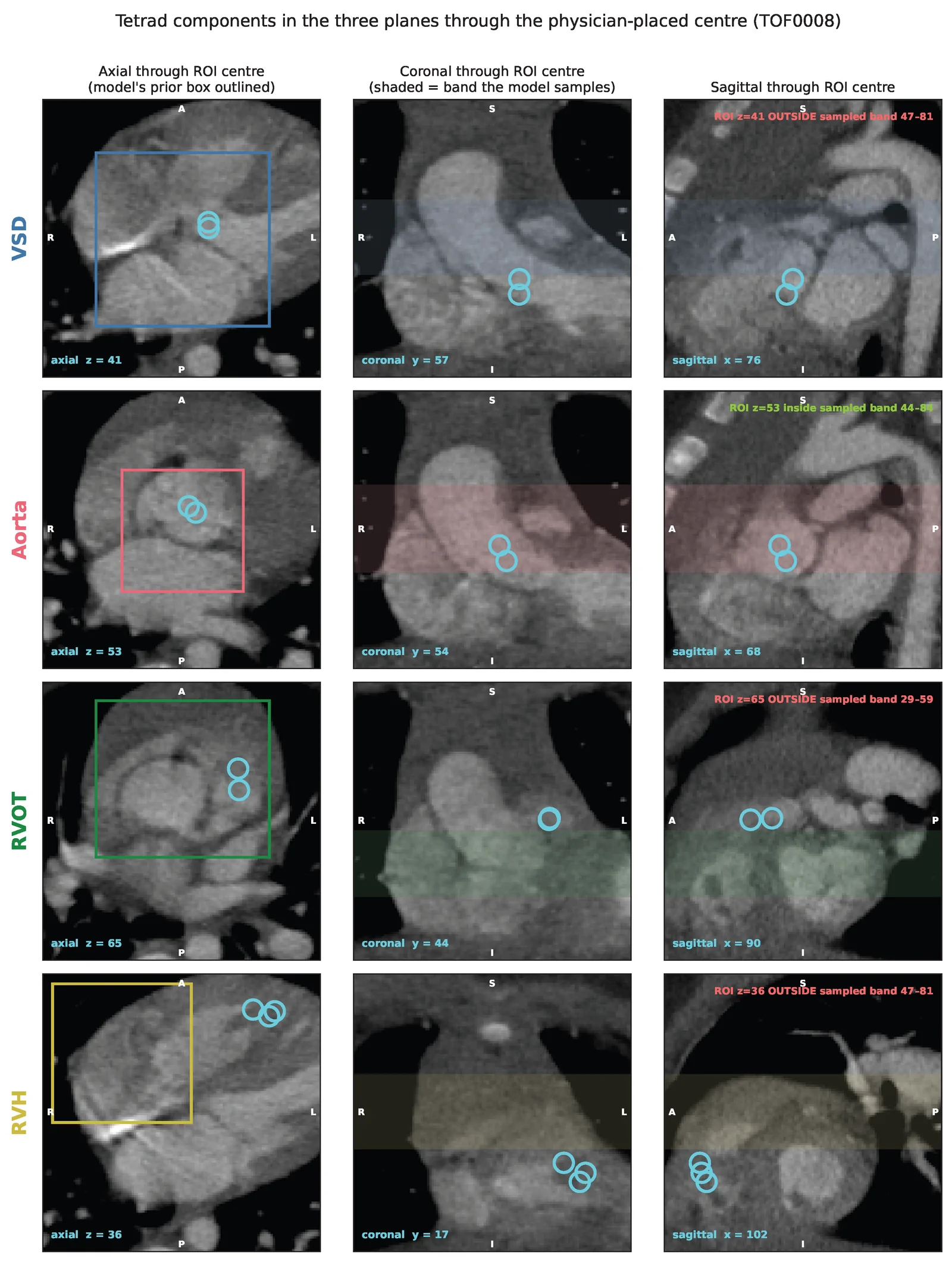
The four fixed priors and the expert-placed targets, shown in the three orthogonal planes through each target’s centre in a representative TOF case. A single axial level cannot display all four components, and the overriding aorta is judged on a long-axis view rather than an axial one, so each row is taken at its own level in the layout the readers used. Coloured outline: The fixed prior box. Shaded horizontal band on the coronal and sagittal views: The z range the model actually samples for that branch, which is the central 30–70% of the region depth rather than the whole box. Cyan markers: The region-of-interest centres placed by the two readers, and for RVH also the reporting radiologist’s independent re-annotation. The aorta target falls inside the sampled band; the VSD, RVOT and RVH targets do not, and the RVH target additionally lies outside the box in the transverse axis. The RVH row should be read with the caveat developed in Section 4.3: hypertrophy is an acquired consequence rather than a diagnostic feature, and at the age of this cohort it is modest and has no validated imaging threshold. Orientation: A: anterior; P: posterior; S: superior; I: inferior; R/L: patient right/left.

### 2.1. Study Design and Ethics

This was a retrospective, single-centre diagnostic-accuracy study of de-identified contrast-enhanced cardiac CT. The study was approved by the Başakşehir Çam and Sakura City Hospital Scientific Research Ethics Committee No. 1 (decision no. 11, 15 January 2025) and conducted in accordance with the Declaration of Helsinki. Because the study was retrospective and used fully de-identified imaging, the committee waived the requirement for written informed consent. The design has one overriding principle, namely to control the confounding intrinsic to TOF imaging, implemented in two stages (acquisition harmonization, then age- and sex-matching) before any model is trained.

### 2.2. Patients and Data Source

Contrast-enhanced cardiac CT was acquired between 2022 and 2026 on a single Canon Aquilion ONE scanner (Canon Medical Systems) at University of Health Sciences Turkey, Başakşehir Çam and Sakura City Hospital, İstanbul. Two cohorts were identified: patients with TOF and controls without structural heart disease. Because contrast-enhanced cardiac CT is not performed on healthy infants, these controls are a clinically selected group of children referred for evaluation of a suspected but subsequently excluded cardiovascular abnormality (for example, an aberrant right subclavian artery or a possible coronary-artery anomaly), whose hearts proved structurally normal, rather than a healthy-population sample; the specific per-patient indications were not systematically tabulated for the matching procedure, a residual (indication) confounder addressed in Section 4.6. All TOF studies were acquired preoperatively, before surgical repair, so the images depict native, unrepaired tetrad anatomy; the model is therefore trained on uncorrected TOF and has no exposure to post-repair appearances. Of 207 source studies (123 control, 84 TOF), 7 contained only a 3D rendered series without a diagnostic volume and were excluded; additional studies lacking a harmonizable series were also excluded.

### 2.3. De-Identification

All DICOM protected health information (name, national ID, birth date, institution, physician names, and private tags) was removed and each patient re-coded. Pixel data were left unchanged. Automated verification covered DICOM headers only (re-coded identifiers, absence of birth date, PatientIdentityRemoved = YES); absence of burned-in identifiers was assessed by visual inspection rather than by an automated pixel-level check. Patient age, sex and study date were deliberately retained, age and sex being required for matching.

### 2.4. Acquisition Harmonization and Cohort Matching

To control confounding, we first restricted the dataset to a single reconstruction (FC15 kernel, 0.5 mm slice thickness, ≥30 slices); where a patient had more than one qualifying series, the series with the most images was taken as that patient’s representative volume, and the remaining qualifying series form the multi-phase set used in Section 3.4. This removed the reconstruction-kernel batch effect that would otherwise separate the groups. We then performed optimal 1:1 matching (Hungarian algorithm [23] minimizing the age difference, with exact sex matching and a 0.5-year caliper) between TOF patients and controls. This yielded 42 matched pairs (84 patients), the analysis cohort for all subsequent experiments.

### 2.5. Preprocessing

Each volume was loaded in Hounsfield units (HU). The heart was localized automatically as the largest “thick” contrast-enhanced blood-pool component using a distance transform (blood pool taken as 150–600 HU and localized on a 4×-downsampled volume, excluding thin great vessels and the abdomen; a 10 mm margin was added on every side and a minimum extent of 60 mm craniocaudally and 55 mm in-plane enforced; and a central-crop fallback was available if no heart-sized component was found, and was not triggered for any patient), cropped and resampled to a fixed $128^{3}$ index grid (each axis scaled independently, so voxel spacing is patient-specific and mildly anisotropic; observed range 0.38–0.87 mm), windowed to [−200, 800] HU, and scaled to [0, 1]. All box definitions and slab positions below are therefore in index rather than physical units. Volume orientation was confirmed as head-first-supine, axial, by inspection of the DICOM headers of the source series.

### 2.6. Tetrad-Component Regions

Rather than feed the whole heart to a single network, we decomposed the standardized volume into four anatomically motivated regional priors, each a fixed box positioned to approximately cover one component of the diagnostic tetrad rather than to delineate it: the VSD (central septal core, $80^{3}$), the overriding aorta (central aortic-axis column, 96×56×56), the RVOT (anterior-superior box, 72×72×80), and RVH (anterior right-ventricular box, 80×64×64) (Figure 2). The intended motivation was twofold: to make the model’s focus interpretable, each branch nominally corresponding to a named anatomical structure, and to inject an anatomical prior that compensates for the small sample. Whether the first was achieved is tested in Section 3.8, and for two of the four branches it was not. Each sub-region is a fixed axis-aligned box defined by constant index ranges within the $128^{3}$ standardized volume, the centre of which coincides with the heart-localization crop centre (Section 2.5); no per-patient re-centring on a blood-pool centroid was applied. In voxel indices [z,y,x], the boxes are as follows: VSD, the central cube ${\left[24:104\right]}^{3}$ (central 62.5% of each axis); overriding aorta, a tall central column z[16:112], y/x[36:92] (96×56×56); RVOT, an antero-superior box z[8:80], y[8:80], x[24:104] (72×72×80); and RVH, an antero-right box z[24:104], y[4:68], x[4:68] (80×64×64). Because TOF distorts cardiac anatomy, most notably through antero-cephalad outlet-septum deviation and aortic over-ride, this fixed geometric prior risks partially missing a displaced target. Box sizes were fixed a priori as generous relative to the expected size of each structure; no formal sizing procedure was applied, and whether this kept each target in view was tested only afterwards (Section 3.8). Axial, coronal, and sagittal QC montages were also generated automatically for every extracted region (one per patient) to enable inspection. No case was excluded on region-quality grounds. The physician annotations used for that audit are axis-aligned regions of interest rather than voxel-level delineations, so containment is quantified by the position and overlap of the annotated region relative to each prior, not by an intersection-over-union against a segmented structure. Two properties of this design bear directly on how the regions should be interpreted. First, the model does not see the whole box: the eight slabs are sampled across the central 30–70% of each region’s depth (Section 2.7), so the z range actually presented to a branch is narrower than its box. Second, whether each box contains its target was audited against physician region-of-interest annotations by mapping them into the model’s index space. The mapping introduces no registration error: the volume given to the annotators and the $128^{3}$ volume used by the model are the same physical crop, both produced by taking the heart bounding box and cutting it with the same region-of-interest operation, differing only in output sampling. Results are reported in Section 3.8. Region definitions here are automatic anatomical priors; a segmentation-based variant would allow coverage to be quantified directly and is left to future work.

### 2.7. Models and the Rationale for a 2.5D Transfer-Learning Design

Two modelling strategies were compared per component. The first is 2.5D transfer learning: an ImageNet-pretrained ResNet18 is applied to three-adjacent-slice axial slabs. The eight slab levels were sampled automatically as evenly spaced *z*-positions across the central 30–70% of the region (no manual, structure-dependent slice selection); each slab was resized to 3×112×112 and ImageNet-normalized, giving ∼8 slabs per patient. The patient-level probability is the unweighted mean of the slab probabilities. The second is a 3D CNN (MONAI DenseNet121) trained on the $64^{3}$-resampled region; both comparator architectures and the three CT-FM configurations are specified in full in Supplementary Table S1. The 2.5D model used Adam [24] (learning rate $1\times 10^{-4}$, weight decay $1\times 10^{-4}$, batch size 16) for 20 epochs; the 3D CNN used Adam (learning rate $1\times 10^{-3}$, weight decay $1\times 10^{-4}$, batch size 8) for 30 epochs, and the full-augmentation 3D re-run used 40. No learning-rate schedule, early stopping or validation-based checkpoint selection was used; the final-epoch weights were taken in every case. The single-phase ConvNeXt-tiny factorial cell used 20 epochs, matching the ResNet18 single-phase baseline, so the backbone increments reported in Section 3.4 are budget-matched; the EfficientNet-B0, attention-MIL and all multi-phase runs used 15 epochs, so the multi-phase increments reported in Section 3.4 confound phase count with training budget and are exploratory only. Both used cross-entropy loss with fixed hyperparameters (no test-set tuning) and light on-the-fly augmentation with ±5% intensity scaling. The 2.5D model applied random flips along both in-plane axes with *p* = 0.5 each; the 3D model applied random flips along all three volume axes with *p* = 0.5 each. Both therefore mirror the patient left–right axis. Laterality-preserving augmentation was not used, although the RVH and RVOT priors are defined by laterality; we did not apply 3D-specific spatial augmentation (random rotation, scaling, or elastic deformation), a limitation revisited in Section 4.5.

The choice of a 2.5D transfer-learning design over an end-to-end 3D network is deliberate and follows the logic established for small clinical cohorts [15]. A 3D CNN must learn volumetric filters from scratch on a few dozen patients; volumetric self-supervised pretraining was introduced precisely because networks trained from scratch on small three-dimensional medical datasets underperform [25]. A 2.5D model instead reuses features already learned by a network pretrained on natural images. The mechanism has been characterized directly: the benefit of ImageNet transfer in medical imaging derives substantially from reuse of low- and mid-level features and from over-parameterization rather than from semantic transfer [26], and feature reuse is the dominant factor when the target dataset is small [27]. That is the regime this study is in.

For aggregating the per-slab probabilities, we used an unweighted mean rather than learned (attention) or max pooling. With only ∼8 slabs per patient and ∼67 training patients per fold, a learned aggregator has very few bags to fit and tends to over-fit, whereas the mean is a zero-parameter, low-variance estimator; max pooling, conversely, is sensitive to a single spuriously high slab. The dilution risk for slice-localized features (e.g., a single VSD-bearing level) is only partly mitigated by restricting slabs to the central 30–70% band. Whether the relevant anatomy falls inside that band was audited against physician annotations (Section 3.8); it does for the aorta (84.3%) but for only 58.9% of VSD, 53.9% of RVH and 0.6% of RVOT annotations. The prediction that a learned aggregator would over-fit at this sample size held empirically: attention-MIL aggregation underperformed the mean (Section 3.4).

Optimization experiments additionally evaluated EfficientNet-B0 and ConvNeXt-tiny backbones, attention-based multiple-instance-learning (attention-MIL) aggregation, and multi-phase data (all FC15/0.5 mm cardiac phases per patient; 218 phase-volumes). We also evaluated a large pretrained CT foundation model (CT-FM, a contrastive encoder pretrained on ∼148,000 CT volumes [28]) in three configurations under the same leak-free folds: (i) frozen global-average-pooled features on our [0, 1] cardiac-windowed volumes; (ii) frozen features on natively preprocessed Hounsfield-unit volumes, re-extracted from the source DICOM with CT-FM’s intensity range ([−1024, 2048]→[0, 1]); and (iii) fine-tuning, unfreezing the encoder’s deepest stage plus a linear head. A handcrafted comparator (gradient-boosted trees) was evaluated analogously on 19 custom features per region: 12 first-order intensity statistics (mean, SD, minimum, maximum, the 10th, 25th, 50th, 75th and 90th percentiles, skewness, kurtosis and first-order energy), a 32-bin intensity entropy, and six grey-level co-occurrence properties (contrast, dissimilarity, homogeneity, grey-level energy, correlation and angular second moment, averaged over distances of one and two voxels and angles of 0 and 90 degrees) computed at 16 grey levels on three axial slices spaced four apart about the region centre. No shape descriptors and no IBSI-standardized three-dimensional texture features were used. All training ran on an Apple-MPS GPU with PyTorch 2.9, MONAI 1.5, and torchvision; random seeds and per-fold splits are fixed in the analysis code.

### 2.8. Hybrid Fusion

Component out-of-fold probabilities were fused by (i) mean and (ii) logistic-regression stacking within the same folds, over all component subsets. The pre-specified primary model is the four-component mean hybrid; best-subset hybrids are reported as exploratory.

### 2.9. Interpretability Analysis

To move beyond a “black-box” classifier and check biological plausibility, we computed Grad-CAM saliency maps [16] (gradients propagated into the final ResNet18 convolutional block, layer4) for each component network and overlaid them on the corresponding CT to inspect whether attention fell on cardiac structures rather than on the body outline or extracardiac tissue. This overlay is qualitative; whether the maps carry more information than a resolution-matched random attribution is tested quantitatively in Section 3.7. Because that test is negative, the primary attribution analysis is occlusion sensitivity [29], computed on the whole-heart baseline model, trained leak-free per fold and applied to its held-out patients. A $24^{3}$ cube was slid over the $128^{3}$ volume on a stride-16 grid (343 positions per patient); at each position the cube was filled with the volume’s own mean intensity, the eight slabs re-extracted and the patient probability recomputed exactly as in deployment. Importance is the fall in predicted probability. Importance at each physician-marked site was compared, over the 42 TOF patients, against the remaining grid cells whose distance from the volume centre lies within 8 voxels, half the stride, of that of the marked cell, in the same patient. The marked cell is the grid cell nearest that patient’s reader-averaged annotation centre. *p*-values are from a 10,000-sample paired sign-flip permutation test, and a sweep over the radius tolerance (0, 4, 8 and 16 voxels) is deposited with the analysis outputs.

### 2.10. Evaluation and Statistics

Evaluation used leak-free, patient-level, stratified five-fold cross-validation. Folds were assigned by patient identifier before any slab or phase extraction, so that every axial slab and every cardiac phase belonging to a given patient resided in the same fold. To gauge the optimism of this single split, we additionally ran 20 repeats of patient-level stratified five-fold cross-validation with the fold assignment re-randomized each repeat (fixed seeds), reporting the mean ± SD out-of-fold AUROC as an optimism-corrected estimate (Section 3.3, Supplementary Table S3). In the multi-phase experiment, the 218 phase-volumes were grouped by patient prior to splitting, preventing any phase of a test patient from appearing in training and thereby avoiding the optimistic bias that per-phase (rather than per-patient) splitting would introduce. The primary metric was the area under the receiver-operating-characteristic curve (AUROC); we also report AUPRC, accuracy, sensitivity, specificity, calibration (slope, intercept, Brier score), and decision-curve net benefit [30]. Ninety-five percent CIs were obtained from a 2000-sample patient-level bootstrap. The corrected-prior contrasts of Section 3.8 were instead tested with a 10,000-resample paired patient-level bootstrap, resampling patients with replacement and recomputing both AUROCs on each resample. Accuracy, sensitivity and specificity are reported at a fixed 0.5 cut-off unless stated otherwise. Paired AUROC comparisons used the DeLong test [31]. Because DeLong is a large-sample normal approximation, each 2.5D-versus-3D contrast was additionally tested without a distributional assumption: under the null that the two models are equally good, the assignment of which model owns which probability is arbitrary within a patient, so a random subset of patients had their two probabilities swapped and both AUROCs recomputed on the full cohort, over 10,000 permutations of the two-sided absolute AUROC difference with an add-one *p*-value. The Benjamini–Hochberg false-discovery-rate family as implemented comprised seven comparisons: the four within-component 2.5D-versus-3D contrasts, the two hybrid-versus-VSD contrasts, and the hybrid-versus-whole-heart contrast. Restricting the family to the four pre-specified component contrasts would give q = 0.019–0.037 for all four; the more conservative seven-member correction is the one reported throughout. Optimized-versus-original comparisons are reported with raw DeLong *p*-values. AUPRC is reported for completeness but is inflated by the artificial 1:1 class balance and would be substantially lower at the true (much lower) TOF prevalence. A whole-heart model (no sub-region decomposition) served as the baseline.

## 3. Results

### 3.1. Cohort and Confounder Control

**Figure 3:**
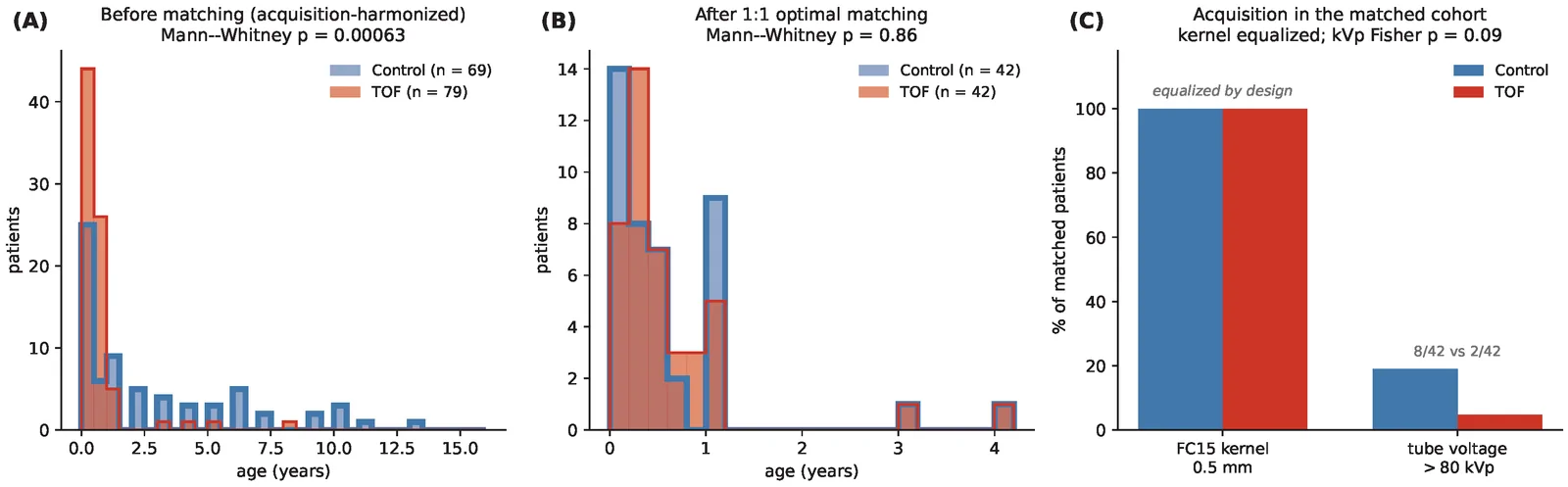
Cohort and confounder control. (**A**) Age distribution in the acquisition-harmonized cohort before matching (69 control, 79 TOF; Mann–Whitney *p* = 6.3×10^−4^). (**B**) After 1:1 optimal age- and sex-matching (42 per group; *p* = 0.86). (**C**) Acquisition in the matched cohort: the reconstruction kernel is equalized by the single-reconstruction restriction, while tube voltage retains a residual imbalance (8 of 42 controls and 2 of 42 TOF above 80 kVp; Fisher *p* = 0.09).

The matched analysis cohort comprised 42 TOF patients and 42 controls. In the full source cohort, the age difference was extreme (control vs. TOF median 5.0 vs. 0.4 years; Mann–Whitney $p=1.7\times 10^{-11}$) and persisted within the acquisition-harmonized cohort ($p=6.3\times 10^{-4}$); matching removed it (0.33 vs. 0.33 years; *p* = 0.86), with balanced sex (*p* = 1.00). Reconstruction kernel was equalized by the single-reconstruction restriction (all FC15, 0.5 mm). Tube voltage was substantially but not completely balanced: 8 of 42 controls and 2 of 42 TOF were scanned above 80 kVp (Fisher *p* = 0.09), a residual acquisition difference not removable by this design. Cohort characteristics are in Table 1; the effect of matching on the age distribution is shown in Figure 3. The full participant flow, from 207 source studies to the 148 acquisition-harmonized studies and then 84 matched patients, with exclusion reasons, is given in Supplementary Table S9. Matching dropped 37 of 79 acquisition-harmonized TOF studies and 27 of 69 controls; the dropped TOF patients were similar in age to those retained (median 0.42 vs. 0.33 years; *p* = 0.31), whereas the dropped controls were markedly older (median 6.0 vs. 0.33 years; *p* = $1\times 10^{-11}$). The matched cohort is predominantly composed of infants (median age 0.33 years, ≈4 months; 40 of 42 patients per group aged one year or younger, of whom 35 TOF patients and 31 controls were under one year; 14 patients had an age recorded as exactly one year, reflecting the whole-year granularity of the DICOM age field above infancy; full range 0–4 years). This narrow age band follows directly from matching. It leaves heart size and acquisition protocol closely comparable between the two groups, which constrains gross body size as a competing explanation for the classifier’s output without eliminating it: morphometric measures alone reach an AUROC of 0.651–0.663 (95% CI 0.53–0.77), above chance but well below the 0.769–0.828 of the deep branches (Section 3.8). It also restricts the cohort to infants, so the findings do not extend to older children or adults (Section 4.6).

### 3.2. Component Classifiers: 2.5D Transfer Learning vs. 3D CNN

The 2.5D transfer-learning model outperformed the 3D CNN for every tetrad component (out-of-fold AUROC): VSD 0.828 (95% CI 0.74–0.91) vs. 0.656; RVOT 0.800 (0.69–0.89) vs. 0.573; overriding aorta 0.786 (0.68–0.88) vs. 0.636; RVH 0.769 (0.66–0.86) vs. 0.596. Across the four components, the 2.5D model showed consistently higher AUROC (DeLong *p* = 0.007–0.037); after Benjamini–Hochberg correction the evidence was significant for three of four components; the RVH comparison did not survive correction (q = 0.065); a paired permutation test (10,000 patient-level swaps of which model owns which probability) gave concordant *p*-values (0.007–0.037; Supplementary Table S10), so the result does not hinge on the DeLong large-sample approximation. We therefore describe this as evidence of, rather than conclusive proof of, superiority. Retraining the 3D CNN with full volumetric augmentation (random rotation, scaling, and elastic deformation) did not close the gap (AUROC 0.57–0.66, no better than the lightly augmented 3D CNN), so the 2.5D advantage is not an artefact of under-regularized 3D training. Per-component metrics are in Table 2.

### 3.3. Hybrid Model and Whole-Heart Baseline

**Figure 4:**
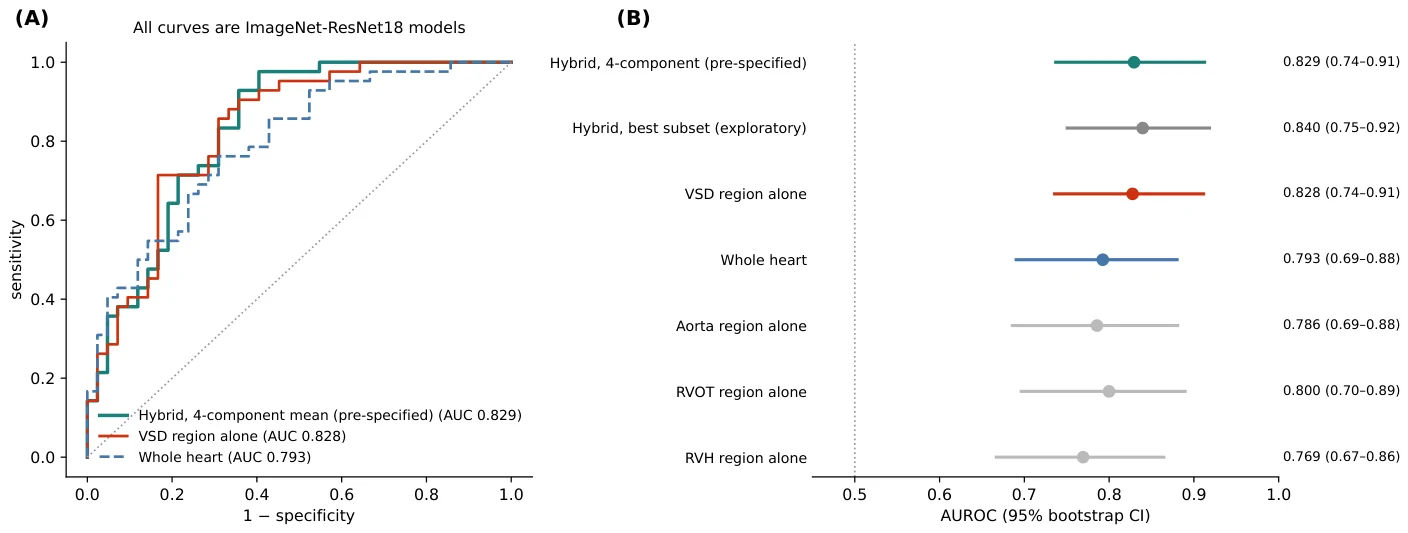
Discrimination of the pre-specified model and its comparators. (**A**) Receiver-operating-characteristic curves for the pre-specified four-component mean hybrid, the VSD region alone, and the whole-heart baseline; all three are ImageNet-ResNet18 models, so the comparison is like-for-like. (**B**) Out-of-fold AUROC with 2000-sample patient-level bootstrap confidence intervals. The pre-specified four-component hybrid is listed first; the best-subset hybrid was chosen on the same out-of-fold predictions and is therefore optimistic.

The pre-specified four-component mean hybrid reached AUROC 0.829 (95% CI 0.74–0.91); the best exploratory subset (VSD + RVOT + RVH) reached 0.840 (0.75–0.92). The whole-heart baseline reached 0.793 (0.69–0.88); the hybrid exceeded it by 0.037, a difference that was not statistically significant (DeLong *p* = 0.32), and the VSD region alone (0.828) matched or exceeded the whole heart (Figure 4, Table 3). The two hybrid-versus-VSD contrasts that complete the multiplicity family were both null (four-component hybrid vs. VSD, Δ = +0.002, DeLong *p* = 0.94, q = 0.94; best-subset hybrid vs. VSD, Δ = +0.012, *p* = 0.64, q = 0.75), as was the hybrid-versus-whole-heart contrast (q = 0.44). The four component probabilities were strongly inter-correlated (Pearson r = 0.56–0.89, highest between the spatially overlapping VSD and aorta regions); consistent with this collinearity, logistic-regression stacking placed the dominant weight on VSD (standardized coefficient +1.0, against +0.48 for RVOT and +0.46 for RVH) and gave the aorta a small negative coefficient (−0.29), an unstable solution typical of correlated inputs, so the pre-specified mean hybrid was both simpler and more robust. Per-fold discrimination varied substantially given the small folds (16–18 patients, 8–9 TOF each): the four-component hybrid ranged from AUROC 0.63 to 0.95 across the five folds (median 0.84) and individual components from 0.53 to 0.98 (Supplementary Figure S1; per-fold values in Supplementary Table S2), consistent with the wide bootstrap CIs. Repeated cross-validation over 20 stratified five-fold splits placed the optimism-corrected hybrid at AUROC 0.811 ± 0.026 (component means 0.74–0.81; Supplementary Table S3), about 0.02 below the single-split estimate, a modest but real optimism.

### 3.4. Optimization

A stronger backbone (ConvNeXt-tiny) with multi-phase data improved every component (VSD 0.857, overriding aorta 0.863, RVOT 0.867, RVH 0.801) and raised the hybrid to 0.884 (four-component) and 0.890 (best subset VSD + aorta + RVOT; 95% CI 0.82–0.95). The optimized best-subset hybrid (VSD + aorta + RVOT) did not significantly exceed the original (raw DeLong *p* = 0.10) or the VSD component alone (*p* = 0.09); the best single component in this configuration was the RVOT (0.867), which was not separately compared. These comparisons lie outside the pre-declared multiplicity family and are reported uncorrected, and the gains fell within the bootstrap CI. The multi-phase configuration also carried unequal phase availability between groups (TOF median 3 vs. control 2 phases per patient; Mann–Whitney *p* = 0.023; Supplementary Table S11), a residual confounder of this exploratory result. Attention-MIL aggregation underperformed mean pooling (0.57), consistent with the small number of training bags.

A full backbone × phase factorial across all four components (Supplementary Table S4) shows that the two levers act unevenly across anatomy. By “region-specific”, we mean, precisely, that the AUROC increment attributable to a given change differs by anatomical region rather than shifting all four branches equally; the increments are given in Table 4. Changing the backbone with single-phase data produced small, broadly similar gains everywhere (+0.007 to +0.029, largest for the VSD). Adding multi-phase data on top was markedly uneven: it contributed +0.071 for the overriding aorta and +0.051 for the RVOT, but +0.019 for RVH and nothing at all (−0.001) for the VSD.

Two structural differences plausibly account for this pattern, and we offer them as interpretation rather than as a tested mechanism. The phases available here are reconstructions of a single contrast-enhanced acquisition at different points of the R–R interval, so they differ in cardiac phase and not in bolus timing; what extra phases supply is variation in chamber geometry and motion state, not in opacification. First, motion and cycle-dependent geometry: The aorta and the RVOT are dynamic outflow structures whose depicted calibre and spatial relationships change through the cardiac cycle, and the phase at which a given structure is best depicted is itself structure-dependent and heart-rate-dependent in children [32]. Sampling several phases raises the chance that at least one reconstruction depicts these two regions at a favourable point of the cycle, rather than committing the model to a single phase chosen for the study as a whole. Second, feature scale: The malalignment VSD is a small, largely myocardial discontinuity at a septal border, where depiction is governed by spatial resolution and partial-volume averaging rather than by cardiac phase [33]; additional phases therefore add redundant rather than complementary information, consistent with an increment indistinguishable from zero (−0.001). RVH sits between these extremes: myocardial wall thickness is itself a function of the cardiac phase at which it is measured [34], so extra phases carry some relevant variation, but the quantity being read is a thickness rather than a lumen. All increments nonetheless remain within the bootstrap CI, so neither lever can be credited with a statistically reliable gain, and this reading should be treated as hypothesis-generating.

### 3.5. Nested Cross-Validation over Model Selection

Repeated cross-validation corrects the variance of a single split but not the optimism of choosing a configuration on the predictions used to report performance. This bias is well characterized: when the same resampling is used both to select a model and to report its accuracy, the reported estimate is optimistic, and nesting the selection step inside an inner loop is the established remedy [35,36]; the effect is largest at the sample sizes typical of proof-of-concept imaging studies [37]. We therefore repeated the analysis with the two selection steps applied to the primary model, the component subset and the decision threshold, moved inside an inner loop (the backbone and phase choices of Section 3.4 were not nested): within each outer training set, a five-fold inner loop selected the component subset (all fifteen non-empty subsets) and the decision threshold, the winning configuration was refitted on the whole outer training set and applied once to the untouched outer test fold; the entire procedure was repeated ten times under different partitions. Pooling the outer-fold predictions gave AUROC 0.787 ± 0.029 across repeats, against 0.811 ± 0.026 for repeated (non-nested) cross-validation and 0.829 for the original single split.

The inner loop did not converge on a single configuration. Across the fifty outer folds, eleven distinct component subsets were selected, the most frequent in only ten of them, and the selected threshold ranged from 0.17 to 0.82 (median 0.42). The subset reported as best-performing on the original single split (VSD + RVOT + RVH) was selected in four of the fifty folds, and the full four-component set was never the inner-loop winner.

Three readings follow. First, the pre-specified primary model is unaffected by any of this, because it involves no selection: the four-component mean hybrid was fixed before analysis, and its estimate stands as reported. Second, at n = 84, the data do not identify a single optimal subset, so a model requiring no selection is the appropriate primary model, and the exploratory best-subset figure of 0.840 (Table 3) should be read as one draw from an unstable selection rather than as an attainable operating performance. Third, together with the component collinearity and the unstable stacking solution (Section 3.3), this indicates that the tetrad decomposition is not an accuracy-improving ensemble; whether it delivers the interpretability it was designed for is tested separately in Section 3.8, where for two of the four regions it does not.

### 3.6. Calibration, Operating Point, and Subgroups

**Figure 5:**
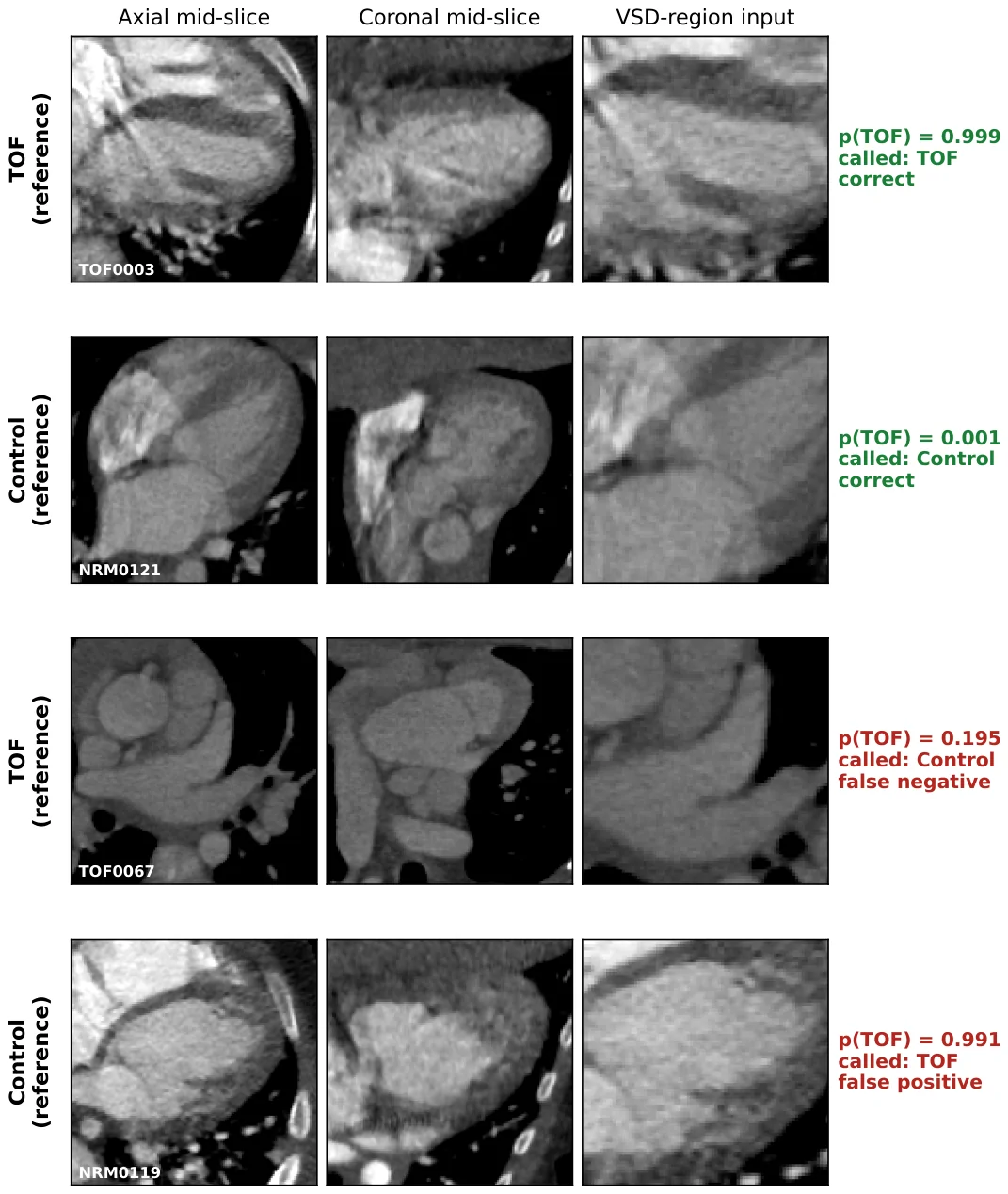
Real cases with the model’s own output, at the out-of-fold Youden operating point (probability 0.37; optimistic, see Section 3.6). One row per patient: Axial and coronal mid-slices of the standardized whole-heart volume, and the VSD-region crop fed to the strongest single component. Probabilities are the four-component mean hybrid taken from the leak-free out-of-fold predictions, so no case shown was in the training set of the model that scored it. All four quadrants of the confusion matrix are represented: a correctly classified TOF (*p* = 0.999), a correctly classified control (*p* = 0.001), a false negative (TOF called control, *p* = 0.195), and a false positive (control called TOF, *p* = 0.991). Both errors are shown alongside both correct classifications.

**Figure 6:**
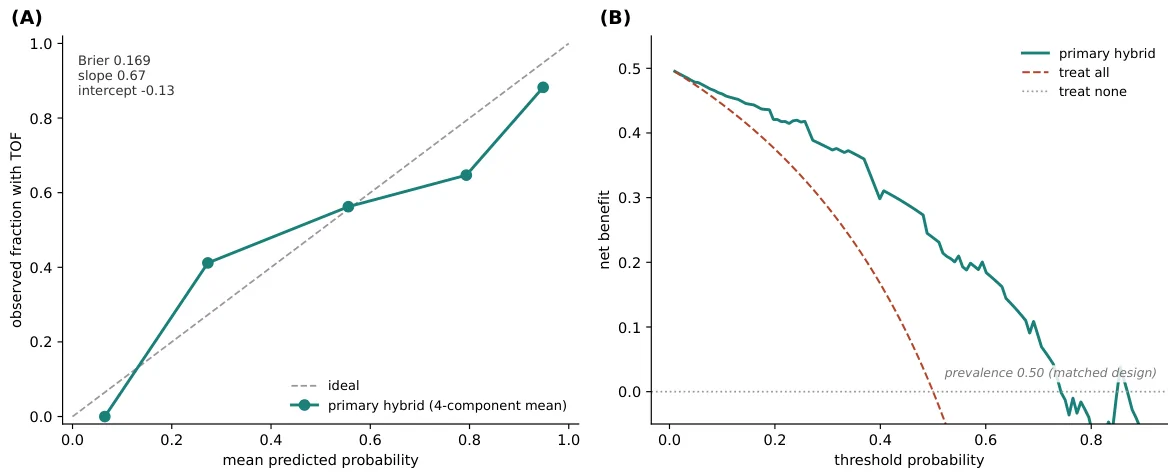
Calibration and clinical-utility analysis for the pre-specified primary hybrid (unweighted mean of the four component probabilities, AUROC 0.829). (**A**) Reliability curve against the ideal diagonal, with the Brier score, calibration slope and intercept. (**B**) Decision curve (net benefit) against treat-all and treat-none. Net benefit is computed at the artificial 50% prevalence of the matched cohort and does not transfer to the true, far lower TOF prevalence [40,41].

The primary hybrid’s calibration slope [38] was 0.67 (2000-sample patient-level bootstrap 95% CI 0.41–1.16), wide enough to include the ideal value of 1, so over-confidence is suggested but not established at this sample size; the Brier score was 0.169, and although temperature scaling improved the slope to 0.89, it did not improve the expected calibration error (ECE 0.097→0.129) or the Brier score (0.169→0.170), so recalibration gave no net reliability gain at this sample size. At the Youden operating point [39] (threshold 0.37), sensitivity was 0.93 and specificity 0.64. Representative cases at that operating point, including one error of each type, are shown in Figure 5. Discrimination was similar by sex (male 0.840, n = 50; female 0.824, n = 34) and by age band (<1 y 0.831, n = 66; ≥1 y 0.792, n = 18), though the older band is too small to support a comparison; per-branch age stratification is given in Section 4.6. Because the operating point and recalibration were both derived on the same out-of-fold predictions, they are in-sample and optimistic. Decision-curve analysis (Figure 6) is shown only for the 1:1 matched cohort (50% prevalence); because net benefit is a function of disease prevalence as well as of discrimination, a curve computed at an artificial 50% prevalence does not transfer to the true, far lower TOF prevalence [40,41]. It should therefore be read as an internal illustration rather than as evidence of clinical benefit.

### 3.7. Feature Interpretation

Grad-CAM maps were evaluated against matched null attributions rather than by visual inspection. Because the body outline is already removed by heart-localization and HU windowing (Section 2.5), central attention is partly built in, so visual plausibility is uninformative here. Grad-CAM at the final ResNet18 convolutional block has a native resolution of 4×4 over the 112×112 slab, that is, sixteen values upsampled 28-fold, so perturbation was applied on a 14×14 grid of 8×8-pixel cells, and no sub-cell interpretation is supportable. Cells were ranked by mean attribution and perturbed in windowed-intensity space before ImageNet normalization, identically across the three adjacent-slice channels; all eight slabs of a patient were perturbed to the same fraction and the eight probabilities re-averaged, exactly as the reported patient probability is formed. Deletion used a constant per-slab mean and insertion a Gaussian-blurred canvas [42]. Each map was compared against two null attributions matched to its own spatial structure: a resolution-matched random map (4×4 noise upsampled identically, averaged over ten draws) and a centred Gaussian blob whose width matched the observed second spatial moment of the maps. Inference is the patient-level paired difference in curve area, with a 2000-sample bootstrap CI and Benjamini–Hochberg correction across the eight region-by-direction comparisons per control.

Against the centred blob, Grad-CAM was superior in all eight comparisons and significantly so in seven (deletion Δ −0.033 to −0.077; insertion Δ +0.053 to +0.084; Supplementary Table S12). The maps therefore carry information beyond the centre prior that preprocessing imposes. Against the resolution-matched random attribution, however, no consistent advantage emerged: Grad-CAM was significantly better in two comparisons (VSD and RVH deletion, Δ −0.029 and −0.032), significantly worse, not merely weaker, in two (aorta deletion Δ +0.029; VSD insertion Δ −0.036), and indistinguishable in the remaining four. For the VSD region, the two removal directions disagreed, with deletion favouring Grad-CAM and insertion favouring the null, a contradiction documented for fixed-value perturbation [43]. At this map resolution, the cell ranking is therefore not reliably more faithful than chance. Two sanity checks were also run [44]. Under cumulative top-down randomization of the network weights, agreement between the perturbed and the trained-model map fell to the random-versus-random floor as soon as the final convolutional block was randomized (Spearman −0.024 against a floor of 0.010) and remained there; under label randomization, with labels permuted within the training folds and every branch retrained, agreement fell from 1.0 to 0.208 ± 0.068. Grad-CAM is therefore sensitive to both the model weights and the labels on this cohort. Randomizing blocks below the final one independently left partially correlated maps (Spearman 0.17–0.27), which is the expected behaviour of a map computed at that depth rather than a failure, since earlier blocks influence it only through the forward pass (Supplementary Table S13).

### 3.8. Region Containment and What the Model Uses

Physician region-of-interest annotations (two independent readers for all four regions in all 84 patients, plus an independent re-annotation of RVH by the reporting radiologist in the illustrative case; per-region counts in Table 5 differ where an annotation was not placed) allow the assumption behind the tetrad decomposition to be tested rather than asserted. Mapping them into the model’s index space shows that the assumption does not hold uniformly (Table 5). Only the overriding-aorta prior reliably presents its target to the model (84.3% of annotation centres inside the sampled band). The VSD prior contains its target in almost all cases (96.3% centre-in-box) but presents it in only 58.9%; the two remaining priors fail outright, RVH presenting its target in 53.9% and RVOT in 0.6%. For the RVOT, 99.4% of annotations lie outside the z band the model actually presents to that branch, a median of 21.6 slices (≈12.5 mm) beyond its edge; only one of the 162 annotations falls inside. For RVH, the failure is in a different axis: the prior spans x 4–68 while the annotation centre lies lateral to it in 55.9% of cases (median x 71.7, range 15–111), so the box is systematically offset toward the midline and contains the marked target in only 43.4% of annotations; in the illustrative case of Figure 2, the two readers’ placements and the reporting radiologist’s independent re-annotation of the RVH target (x 96.5, 103.9, 106.5) agree with one another to within 5.4–11.9 voxels in three dimensions, and all three fall outside the box. Per-reader centre-in-box containment agrees to within 4 percentage points for every region; per-reader in-band containment differs by up to 10.5 percentage points (VSD).

**Figure 7:**
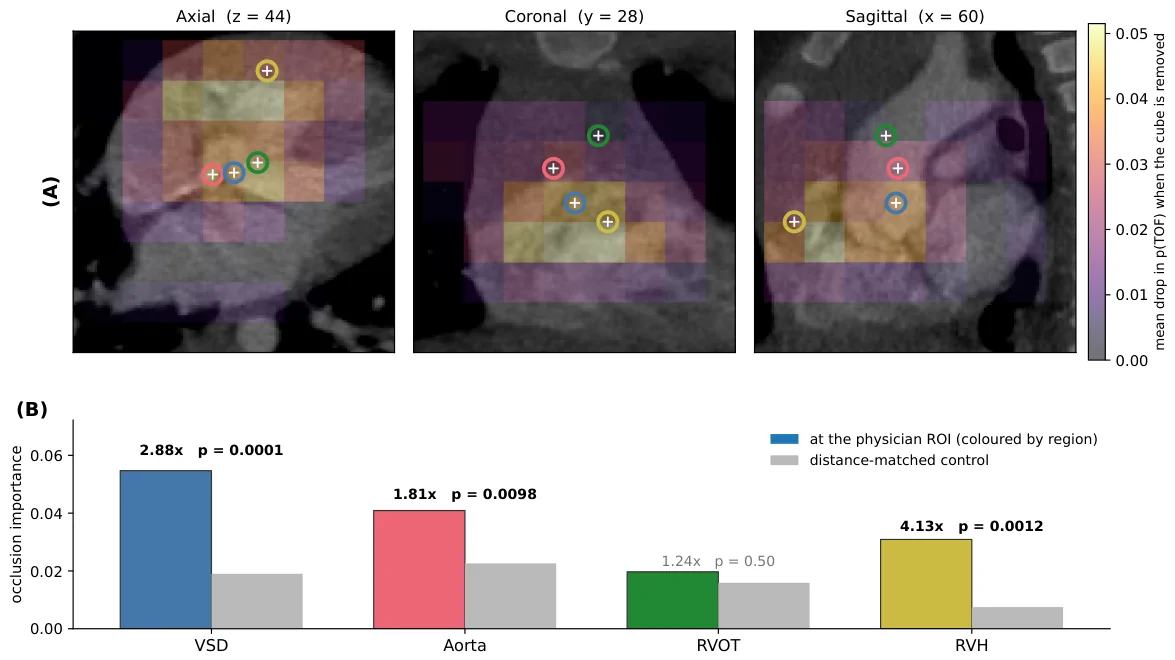
Occlusion sensitivity: Which parts of the heart the model actually uses. All panels are computed on the whole-heart baseline model (AUROC 0.793), which takes the entire standardized volume as input and uses no regional prior, over the 42 TOF patients. (**A**) Group-mean map, shown in the three orthogonal planes through its peak, overlaid on a representative case. Warmer colour marks a larger fall in the predicted probability when a $24^{3}$ cube centred there is removed and the patient probability recomputed. Circles mark the cohort-median physician annotation centre for each component. (**B**) Importance at each physician-marked site against a control drawn from grid cells lying within 8 voxels of that site’s own radius from the volume centre, in the same patient; this control is necessary because all four targets lie near the centre of the heart, where importance is higher regardless of anatomy. Ratios and permutation *p*-values are given above each pair. The model relies on the septal, aortic and right-ventricular sites well above the matched control (2.9×, 1.8× and 4.1×) and shows no preference for the RVOT site (1.2×, *p* = 0.50). This analysis is preferred to a gradient-based saliency map: it is causal; its resolution is set by the cube size rather than by the network stride; it avoids the known tendency of gradient-based maps to highlight locations outside the region actually driving the prediction [45]; and it does not depend on a saliency assumption that the faithfulness audit (Section 3.7) could not support.

Two further analyses ask what the model uses instead. First, a purely geometric baseline: Twenty global shape and density descriptors computed on the standardized volume, with no learning, reached AUROC 0.651 (logistic) and 0.663 (gradient boosting) under the same folds, against 0.769–0.828 for the deep branches and 0.829 for the hybrid. Gross size and shape therefore account for part but not most of the discrimination. Second, occlusion sensitivity [29] applied to the whole-heart baseline model (AUROC 0.793), which uses no regional prior (Figure 7, Table 6): Removing a $24^{3}$ cube and recomputing the patient probability shows it relies on the physician-marked VSD, aortic and RVH sites 2.88, 1.81 and 4.13 times more than tissue at the same radius from the volume centre in the same patient (permutation *p* = 0.0001, 0.0098 and 0.0012). The RVOT site shows no such preference (1.24 times, *p* = 0.50). These are computed over the 42 TOF patients; over all 84, the three ratios strengthen (4.51, 2.51 and 4.70) and the RVOT importance becomes slightly negative, so the RVOT null does not depend on the restriction. The whole-heart baseline therefore responds to tissue at three of the four physician-marked locations more than to distance-matched tissue, and the one location it does not favour is the one whose regional prior misses its target most severely.

Finally, we asked whether correcting the priors recovers anything (Table 6). Because the readers annotated knowing the diagnosis, per-patient annotation coordinates cannot enter the model: the twelve coordinates alone, with no image, separate TOF from control at AUROC 0.931. We therefore moved each box by a single global offset, taken from the median annotation of the training controls only and recomputed within each fold, so that neither a held-out patient nor any TOF anatomy informs the box it is later cropped with. No region changed significantly: RVOT +0.083 (*p* = 0.10), aorta +0.027 (*p* = 0.32), RVH +0.010 (*p* = 0.86), and VSD −0.031 (*p* = 0.46); every 95% CI of the paired difference crosses zero. The priors were demonstrably mispositioned, and repositioning them did not alter discrimination. Both available forms of re-centring were tried. That offset is the strongest correction that carries no per-patient label information, and it changed nothing. Centring each patient’s crop on that patient’s own annotation does raise the reported figures, but not usably: because the readers annotated knowing the diagnosis, those coordinates are themselves a label, and we report that experiment as invalid rather than as a result. A leak-free per-patient crop would have to be centred on an automatically derived landmark rather than on an expert mark, which requires the segmentation step left to future work.

A repositioning derived from expert annotations could itself be mis-specified, so we ran the control that removes anatomy from the question altogether: boxes of the same sizes placed at random positions inside the standardized heart, five independent placements per size, each applied identically to every patient and otherwise trained through the same pipeline. Across the twenty placements, the mean AUROC was 0.765 (SD 0.036, range 0.667–0.823), against 0.796 for the four published priors and 0.793 for the whole-heart baseline. The direction is consistent: only three of the twenty random placements exceeded the published prior of matching size, and every one of the twenty exceeded the morphometry-only ceiling of 0.663. But the advantage averages just 0.030, and with five draws per region, no formal test, and a bootstrap CI half-width of about 0.085, this cohort cannot establish that it is real.

### 3.9. Robustness (Ablations)

Discrimination was insensitive to the choice of feature extractor and architecture. A handcrafted radiomics model (XGBoost) was weaker per component and degraded the fusion when added to the exploratory multi-phase ConvNeXt hybrid (0.884 → 0.850; radiomics-only 0.731). A large pretrained CT foundation model (CT-FM [28]) did not beat the matched 2.5D hybrid in any configuration: the frozen-feature hybrid reached 0.748 with our cardiac-windowed proxy, improved to 0.779 with native Hounsfield-unit preprocessing, and was 0.769 after fine-tuning the encoder’s deepest stage (Supplementary Table S6), all below the 2.5D hybrid. The 3D CNN (both light and full augmentation) and attention-MIL also underperformed. With CT-FM’s native preprocessing and fine-tuning, and with the 3D CNN re-trained under full volumetric augmentation, no alternative significantly exceeded the 2.5D hybrid (Table 3; Supplementary Tables S5 and S6); at this cohort size the comparison bounds rather than settles the architecture question.

## 4. Discussion

This study applies deep learning to tetralogy of Fallot on cardiac CT under explicit control of the confounding that would otherwise dominate the comparison. The anatomy-guided, confounder-matched pipeline separated TOF from matched controls with an out-of-fold AUROC of 0.829, corrected to 0.811 by repeated cross-validation. Deep learning has been applied to congenital heart disease on CT before, at comparable and at far larger scales [7,8,9]; what has not been done is to control the confounding intrinsic to the comparison. Our design differs from the prior binary TOF classifier [10] in three respects that bear on validity: matching and harmonization remove the measured age difference and equalize the reconstruction kernel by design, although the resulting change in performance was not quantified against an unmatched comparator (Section 4.6); cross-validation is strictly patient-level; and the component subset and decision threshold are additionally re-run inside an inner loop, placing the estimate at 0.787 ± 0.029 (Section 3.5); the primary figures reported here are not themselves nested.

The VSD region alone matched the whole heart, and hybrid gains over the whole-heart baseline were modest and non-significant. At this sample size, therefore, a single central sub-volume carries as much discriminative information as the whole heart, without identifying which anatomy within it is responsible. If one region carries the signal, is the four-component decomposition justified? On accuracy alone, no: the pre-specified hybrid (0.829) barely exceeded VSD alone (0.828), the components were strongly collinear (Section 3.3), and nested cross-validation showed that no single subset is reliably identifiable at this sample size (Section 3.5). Its intended value was interpretability, a per-component map a radiologist could read against the diagnostic tetrad, but the containment audit shows that value was not realized here; what survives is a scaffold for the planned physician-annotated, spatially non-overlapping regions. Our own audit shows why the decomposition did not deliver it here: two of the four priors do not contain the structures they are named after (Section 3.8), so the branches were never reading four distinct tetrad components.

### 4.1. Comparison with the Literature

Table 7 places our result against the published literature. The mechanisms behind the spread matter more than the numbers themselves.

The dominant driver is the unit of analysis. The only prior binary TOF-versus-control classifier on cardiac CT [10] reported AUC 0.96 but was cross-validated on 80 radiologist-selected slices drawn from 20 children: with four slices per child, essentially every test slice had a same-patient sibling in training, and manual pre-selection of diagnostic slices is an oracle unavailable at deployment. The size of this effect is directly measurable: evaluating one foetal-echocardiography model under intra- and then inter-patient splitting, with nothing else changed, moved mean average precision from 98.3% to 82.4% [48]. Such figures characterize fold-to-fold stability under leakage rather than generalization.

The second driver is cohort size. The realistic envelope for congenital diagnosis on CT is set by much larger work: 86.0% accuracy across seventeen classes in more than 3750 patients, comparable to junior cardiovascular radiologists [7]. The ImageCHD baseline reaches 72.5% overall under full prediction (82.0% under selective prediction at 88.4% coverage) and, for TOF specifically, classifies 7 of 12 cases correctly while assigning 4 to isolated ventricular septal defect [8]. This is the confusion a component-wise representation was intended to expose; the containment audit shows that this intention was not realized here (Section 3.8). Instead, it shows an independent argument for a component-wise rather than a whole-heart representation. These multi-class accuracies are not directly comparable to a balanced binary AUROC; we note them to indicate the scale at which CT-based congenital diagnosis has been attempted, not as a benchmark for our task.

The third is how the comparison group is constituted. The current benchmark for TOF diagnosis is echocardiographic, at AUROC 0.989 across four centres [46], achieved against a comparator that included an explicit “TOF mimics” class (480) alongside 1018 healthy controls, rather than healthy controls alone. That choice is an acknowledgement, from the opposite methodological direction to ours, that the control group governs what such a figure means. CT-based TOF diagnosis under any form of confounder control remains unaddressed.

Our values are otherwise unremarkable for small single-centre imaging AI: single-split estimates carry an optimism that repeated cross-validation partly corrects and nested cross-validation addresses more fully [37], and error bars near ±10% are inherent at n≈100, with the across-fold standard error underestimating them [50]. The contribution is a confounder-controlled internal estimate rather than a maximal AUROC.

### 4.2. Clinical Implications and the Nature of the Task

A critical question for translation is what an internal AUROC near 0.81–0.83 means in practice, and whether the task represents a clinical need at all. On contrast-enhanced cardiac CT, established TOF is rarely a diagnostic dilemma: the malalignment VSD, overriding aorta and outflow obstruction are evident to the reading radiologist, and echocardiography, the diagnostic reference standard, has usually established the diagnosis already [2,11]. We therefore claim no standalone diagnostic indication. In congenital heart disease, the purpose of CT is rarely to assign a label but to characterize anatomy in the detail that surgical and transcatheter planning require: coronary origin and course, pulmonary-artery morphology, the right-ventricular outflow tract, arch configuration, and systemic-to-pulmonary collaterals. It is these features, not the diagnostic category, that drive surgical complexity [4,12].

Read in this light, the work is a controlled test of how to learn from the small, heterogeneous datasets intrinsic to paediatric cardiac CT, where data scarcity is a structural feature of the field rather than a temporary inconvenience; TOF serves as a well-defined benchmark, and the longer-term goal is an anatomy-aware encoder supporting segmentation, quantitative vascular measurement, and surgical phenotyping.

Any nearer-term use would be automated quality control or a pre-read flag for triaging studies where paediatric cardiac expertise is not immediately available, rather than autonomous diagnosis, and even that requires care with the operating point. Sensitivity (0.93) and specificity (0.64) were measured at the artificial 50% prevalence of the matched cohort; at the true, far lower prevalence of TOF among scanned children, the positive predictive value would be low and the number of false-positive flags large in absolute terms. Low prevalence therefore makes false positives more, not less, of a burden, the opposite of what raw specificity suggests, so any deployed threshold and any claim of net benefit must be re-established at realistic prevalence on external data. We view the eventual role of such systems as augmenting, not replacing, expert radiological interpretation.

### 4.3. The Tetrad Components Are Not Diagnostically Equivalent

Our own audit shows that two priors miss their targets and that repositioning them changes nothing. The clinical literature bears on how this failure should be interpreted, and it also revises the premise on which the decomposition was built.

The four eponymous features are not four independent malformations. Contemporary morphological work identifies antero-cephalad deviation of the outlet septum, with abnormal septoparietal trabeculations, as the hallmark of the condition [1,51]; that single deviation simultaneously produces the malalignment interventricular communication, the aortic over-ride and the subpulmonary obstruction. Right-ventricular hypertrophy is different in kind: it is described as the haemodynamic consequence of those lesions [1], an acquired response to chronic systemic-level right ventricular pressure [11]. Three components carry the diagnosis; the fourth reflects the duration of the resulting pressure load.

This distinction is decisive at the age of our cohort. Because foetal shunts equalize ventricular afterload, a hypertrophied right ventricle is not a prenatal feature and the two ventricles have similar wall thickness in utero [52]; hypertrophy begins after birth and its magnitude increases with age [53]. It is superimposed on a neonatal baseline that is itself relatively right-dominant, with newborn right ventricular mass index about 20% above adult values and left ventricular mass index about 30% below, giving a right-to-left mass ratio roughly 75% higher than at maturity [54]; over the first four months that ratio falls steeply toward adult values as right ventricular mass index decreases and left ventricular mass index rises [55]. Our matched cohort has a median age of 0.33 years, with most patients under one year (Section 3.1), so the RVH branch was asked to detect a feature that is modest at this age, superimposed on physiological right-ventricular prominence, and for which no validated infant threshold exists on echocardiography, let alone on CT [56]. On cross-sectional imaging, the reported diagnostic and surgical-planning targets are septal malalignment, over-ride, outflow-tract and pulmonary arterial anatomy, and coronary supply; hypertrophy is described but not used as a criterion [2,12].

There is direct precedent for encoding this asymmetry. An echocardiographic deep-learning detector, in a cohort of comparable age, classified a study as TOF on three components and excluded RVH from its diagnostic criteria because it may or may not be present depending on when imaging was performed [57]. Our design did not make that distinction, and treating four unequal features as four equivalent branches is a design error independent of where the boxes were placed. It also explains the collinearity we measured between the component probabilities, beyond the spatial overlap of the boxes: the features share a morphogenetic origin and a haemodynamic consequence, and the two contributions cannot be separated in this cohort, so a decomposition into four branches could not have yielded four independent views.

### 4.4. Anatomical Plausibility and Interpretability

Two lines of evidence bear on what the model uses, and they should be read in order of strength. The stronger is occlusion sensitivity (Section 3.8; Figure 7), which is causal: removing tissue at the physician-marked septal, aortic and right-ventricular sites moves the decision 1.8 to 4.1 times more than removing tissue at the same radius from the volume centre in the same patient. The RVOT site showed no such preference (1.24, *p* = 0.50), the one location whose prior misses its target most severely. Gross morphometry alone reaches only 0.651–0.663, so heart size and shape account for part but not most of the discrimination, and the occlusion evidence indicates that the whole-heart baseline reads cardiac anatomy beyond gross morphometry. The weaker line is the saliency analysis. Quantifying the Grad-CAM maps (Section 3.7; Supplementary Tables S12 and S13) supports two claims and refuses a third. The maps are sensitive to both the model weights and the training labels, so they are not the input-dependent edge detectors that saliency methods are sometimes shown to be. The widely cited failure result applies to Guided Backpropagation and Guided Grad-CAM rather than to Grad-CAM, which was sensitive to both randomizations in the original report [44]; sensitivity is a necessary condition, not evidence of faithfulness. Grad-CAM was superior to a centred blob of matched width in all eight comparisons and significantly so in seven, so the maps carry spatial information beyond the centre prior that heart-localization and HU windowing impose, though what anatomy that information corresponds to is not established, a concern the preprocessing itself raises. But Grad-CAM did not consistently beat a resolution-matched random attribution, and we therefore make no localization claim. The explanation is probably structural rather than specific to this cohort: at the final convolutional block, the map has sixteen degrees of freedom over the whole region, so a single cell spans roughly a quarter of each axis, coarser than a malalignment VSD in an infant heart. Coarse class-activation maps are known to lose findings at this scale [58], the gap between saliency and expert localization widens as the target gets smaller [18], and of the eight methods evaluated in medical imaging, none satisfied every trustworthiness criterion [17]. Because ResNet18 pools globally before its classifier and the map is taken at the last convolutional layer, our Grad-CAM is moreover mathematically identical to CAM there [16], so CAM itself would give the same numbers; finer variants computed at earlier layers [58] were not evaluated. Faithfulness is in any case not correctness: a perfectly faithful map of a model keying on a residual indication cue would look no different [13]. We accordingly present the maps as illustrative, noting only that the highest-performing branch is the one whose prior is centred on the septum, which the audit shows is also the prior that most often contains its named target, though not the one that most often presents it (Section 3.8). Localization proper requires the voxel-level expert annotation discussed in Section 4.7.

### 4.5. Robustness to Modelling Choice

The conclusion was robust to model capacity. Neither a stronger backbone with multi-phase data, a handcrafted radiomics model, nor a large pretrained CT foundation model [28], the last tested with native Hounsfield-unit preprocessing and with fine-tuning, significantly improved on the matched 2.5D pipeline, and several degraded it. These are configuration-specific comparisons: each alternative was tuned for a fair match but not exhaustively, so they bound what these models achieve under comparable effort rather than their best attainable performance. At a bootstrap CI half-width of about 0.085, this cohort cannot resolve differences of the size observed (up to +0.06); architecture is untested here rather than ruled out. This is not an isolated observation: on non-contrast cardiac CT, handcrafted radiomic features have likewise outperformed both CT-FM and RadImageNet embeddings for coronary-calcium classification [59]. One caveat tempers the 2.5D-versus-3D comparison: the 3D CNN received only light augmentation. Retraining it with full volumetric augmentation for 40 epochs, a larger budget than the 30 used for the lightly augmented 3D CNN, did not narrow the gap (Section 3.2), so the 2.5D advantage is robust to 3D regularization rather than an augmentation artefact. This applies to the 2.5D-versus-3D contrast specifically, at this sample size only; a far larger cohort could change the balance.

### 4.6. Limitations

This study is single-centre, retrospective, and modest in size (n = 84), so confidence intervals are wide and the optimization gains, consistent in direction but not statistically significant, fall within them. At 42 events, the achieved precision is a bootstrap 95% CI half-width of about 0.085 AUROC, adequate to demonstrate above-chance discrimination, not to resolve small differences between competing models. The model has correspondingly limited exposure to the anatomical and acquisition variability of the wider TOF population (post-repair anatomy, diverse collateral patterns, other scanners and contrast protocols), and there is no external validation; the reported performance is an internal estimate, not an estimate of real-world accuracy.

Three design choices bound interpretation. The component regions are automatic anatomical priors that overlap spatially, and the audit in Section 3.8 shows that two of them do not contain the structures they are named after: the RVOT prior contains its target in only 48.1% of annotations and the band the model actually samples excludes it in 99.4%, while the RVH prior sits medial to its target, containing it in only 43.4% of annotations. The regions should therefore be read as fixed sub-volumes of the standardized heart rather than as anatomical components, and the tetrad framing as a design intention rather than a verified property. Repositioning the priors did not change discrimination, so this mislabelling does not affect the reported numbers, but it removes the anatomical justification for the decomposition. The calibration slope was 0.67 with a confidence interval that includes 1 (0.41–1.16), so over-confidence is suggested but not established; temperature scaling raised the slope to 0.89 without improving expected calibration error or Brier score, and the raw scores should not be read as individual risks: acceptable for a triage rank, a real limitation for any quantitative probability. And matching maximizes internal validity at the cost of generalizability to unmatched, all-ages populations.

Right-ventricular hypertrophy is not diagnostically equivalent to the other three components. It is an acquired consequence of the primary lesions rather than a diagnostic feature in its own right [1,11]; it develops after birth and grows with age [52,53], and it is superimposed on the physiological right-ventricular prominence of infancy [54,55]. At this cohort’s age (Section 4.3), hypertrophy is modest, and no validated infant threshold exists on echocardiography [56], and we are aware of none on CT. The data support this reading only partially. Stratifying by median age, every branch discriminated numerically better in the older half (VSD 0.806 to 0.845, aorta 0.739 to 0.804, RVOT 0.721 to 0.835, RVH 0.727 to 0.799), differences well inside the bootstrap CI, so no branch-specific age effect can be demonstrated and the direction should not be interpreted; within TOF patients, age did not correlate with predicted probability for any component. The clinical argument for demoting RVH rests on the described physiology rather than on a differential demonstrable in this cohort.

Two residual sources of bias deserve emphasis. The controls were referred for evaluation of a suspected but excluded cardiovascular abnormality outside the cardiac chambers and septa (for example, an aberrant right subclavian artery or a possible coronary-artery anomaly) and had structurally normal hearts; this makes them a well-defined “structurally normal heart” comparator, but indication itself was not matched, so the model may in part separate TOF from these other reasons for imaging rather than from the general population. And because every TOF study was preoperative and none of these patients had undergone any intervention, the model has seen only native, unrepaired anatomy; surgical or catheter-related change cannot contribute to the discrimination, but neither has the model any exposure to post-repair appearances, which is what a deployed system would most often encounter.

Finally, several optimistic biases compounded on the original single split, where the operating point, the recalibration mapping and the best-performing component subset were all chosen on the predictions used to report performance. Repeated cross-validation corrects the split variance but not that selection optimism; nested cross-validation, which moves every such choice inside an inner loop, is reported in Section 3.5. None of these corrections addresses single-centre sampling, spectrum bias, or scanner-specific signal, which require external validation.

Two limitations specific to the region audit deserve statement. First, the physician annotations were made with the diagnosis known, so they can be used to ask a geometric question, namely whether this box contains that structure, but not as a model input: the twelve annotation coordinates alone separate the groups at AUROC 0.931 with no image at all, and an experiment that centred each patient’s crop on that patient’s own annotation produced correspondingly inflated values, which we therefore do not report as results. Any future use of expert region annotations in this cohort requires blinded reading. Second, at n = 84, the bootstrap 95% CI half-width is about 0.085, so the largest change we observed after repositioning a prior, +0.083 for the RVOT, cannot be resolved by this cohort. The correct statement is that no difference could be demonstrated, not that none exists.

### 4.7. Future Directions

The decisive next step is external, multi-centre, and prospective validation. Two internal analyses would further bound what is reported here: evaluation of the larger, unmatched cohort with propensity-score or inverse-probability weighting, to probe the internal- versus external-validity trade-off directly; and voxel-level expert segmentations, which would supply the ground truth that the localization axis of interpretability requires but this analysis lacked. Such annotation must be collected with the annotators blinded to the diagnosis: the coordinates of the annotations available here carry enough diagnostic information on their own to contaminate any model that consumes them, which is a constraint on study design rather than on the annotators. Multi-phase and, eventually, multi-modal inputs are natural extensions once a larger cohort is available, as deep-learning fusion of multiple views and modalities has already proven feasible for detecting congenital heart disease [60].

A second direction is the initialization itself. Radiology-specific and volumetric in-domain pretraining can outperform ImageNet, with the largest reported gains precisely in the low-sample regime that characterizes this study [25,28,61,62]. The evidence is not unidirectional: ImageNet transfer remains a competitive baseline, reaching expert-level accuracy even in cardiac imaging [63], and feature reuse contributes materially to its success [27], while standard ImageNet architectures are over-parameterized for medical tasks, lightweight models perform comparably, and part of the apparent gain is feature-independent weight scaling rather than semantic transfer [26], and radiology-pretrained weights have underperformed ImageNet on other small radiographic tasks [64], so the optimal pretraining source is task- and domain-dependent rather than universally medical, consistent with our own finding that neither CT-FM nor handcrafted radiomics improved on ImageNet initialization here. Handcrafted texture descriptors paired with a compactly optimized classifier likewise remain a competitive baseline in medical imaging—on coronary CT angiography, radiomic features with a gradient-boosting classifier have matched or exceeded deep learning for plaque and stenosis characterization [65]; our radiomics-plus-gradient-boosting ablation follows that design philosophy, and its underperformance indicates that the discriminative signal for TOF in this cohort is not adequately captured by predefined texture statistics. Two considerations are specific to this setting. All available CT foundation models are pretrained on predominantly adult data, and adult-trained CT models degrade substantially in children [66]; and we are not aware of any pretraining corpus or foundation model built for paediatric cardiac CT or congenital heart disease. Systematically benchmarking paediatric-adapted, in-domain pretraining against ImageNet initialization is therefore a well-defined open question rather than a settled recommendation (effect sizes in Supplementary Table S14), and one we intend to address.

Beyond classification, the principal clinical target is surgical-planning phenotyping: extracting the anatomical features that actually govern operative decisions in TOF, namely coronary-artery origin and course across the RVOT, pulmonary-valve annulus dimensions, and pulmonary-artery branch anatomy, for which the present anatomy-guided, confounder-controlled encoder is intended as methodological groundwork rather than a finished tool.

## 5. Conclusions

We developed and internally evaluated a confounder-matched, anatomy-guided deep-learning pipeline that recognizes tetralogy of Fallot on cardiac CT, reaching a repeated-cross-validation internal AUROC of 0.811 ± 0.026 and 0.787 ± 0.029 once model selection was nested (single-split point estimate 0.829; an exploratory optimized variant reached 0.890 but was selected on out-of-fold data and did not significantly exceed the primary model). Grad-CAM maps were not reliably more faithful than a resolution-matched random attribution, so no localization claim was made. Two of the four sub-volumes did not contain the tetrad component they were named for, repositioning them did not change discrimination, and randomly placed boxes lost only 0.030 on average, so the anatomical placement of the priors could not be shown to matter and the tetrad framing was a design intention rather than a verified property. To our knowledge, this is the first CT-based, confounder-controlled deep-learning analysis in TOF. Increasing model capacity did not significantly improve on the matched pipeline; the separate contribution of matching itself was not isolated against an unmatched comparator and remains to be quantified. The task itself is not a clinical need: in a patient referred for cardiac CT, the diagnosis is already established by echocardiography, and the scan is performed to delineate anatomy rather than to detect the lesion. This work is therefore a methodological baseline for confounder-controlled learning on paediatric cardiac CT, not a diagnostic aid, and it produces none of the anatomical measurements that operative planning requires. As a proof of concept on a small, single-centre cohort without external validation, it establishes feasibility and an optimistic internal estimate rather than clinical readiness; the findings are hypothesis-generating, and external, multi-centre, prospective validation is the essential next step before any clinical use.

## Figures and Tables

**Table 1:** Matched-cohort characteristics (n = 84). Age was compared by Mann–Whitney U and sex by Fisher exact test.

Characteristic	Control (n = 42)	TOF (n = 42)	*p*
Age, years, median (IQR)	0.33 (0.17–0.92)	0.33 (0.27–0.73)	0.86
Age, years, mean ± SD	0.60 ± 0.74	0.60 ± 0.73	—
Age, years, range	0.01–4.0	0.00–4.0	—
Sex, M/F	25/17	25/17	1.00

Before matching, the acquisition-harmonized cohort (n = 148) differed in age (Mann–Whitney $p=6.3\times 10^{-4}$); matching balanced age and sex. Single scanner (Canon Aquilion ONE), contrast-enhanced, FC15 kernel, 0.5 mm.

**Table 2:** Per-component discrimination (out-of-fold, n = 84).

Component	Model	AUROC (95% CI)	AUPRC	Sens	Spec
VSD	2.5D (ImageNet)	0.828 (0.74–0.91)	0.808	0.81	0.69
VSD	3D (DenseNet121)	0.656 (0.53–0.78)	0.651	0.62	0.67
VSD	ConvNeXt + multi-phase	0.857 (0.77–0.93)	0.861	0.71	0.76
Aorta	2.5D (ImageNet)	0.786 (0.68–0.88)	0.767	0.76	0.69
Aorta	3D (DenseNet121)	0.636 (0.51–0.76)	0.636	0.60	0.64
Aorta	ConvNeXt + multi-phase	0.863 (0.78–0.93)	0.846	0.76	0.76
RVOT	2.5D (ImageNet)	0.800 (0.69–0.89)	0.759	0.81	0.67
RVOT	3D (DenseNet121)	0.573 (0.46–0.70)	0.568	0.71	0.40
RVOT	ConvNeXt + multi-phase	0.867 (0.79–0.93)	0.879	0.81	0.67
RVH	2.5D (ImageNet)	0.769 (0.66–0.86)	0.776	0.67	0.71
RVH	3D (DenseNet121)	0.596 (0.47–0.72)	0.562	0.52	0.69
RVH	ConvNeXt + multi-phase	0.801 (0.70–0.90)	0.787	0.79	0.69

VSD, ventricular septal defect; RVOT, right-ventricular outflow tract; RVH, right-ventricular hypertrophy. The 2.5D model exceeded the 3D CNN for every component (DeLong *p* = 0.007–0.037). ConvNeXt + multi-phase rows are exploratory: they were trained for 15 rather than 20 epochs, and phase availability differed between groups (Supplementary Table S11).

**Table 3:** Hybrid models, whole-heart baseline, and ablations (out-of-fold, n = 84).

Model	AUROC (95% CI)	AUPRC	Sens	Spec
Hybrid 4-way (ResNet18)	0.829 (0.74–0.91)	0.806	0.79	0.69
Hybrid best (VSD+RVOT+RVH, ResNet18)	0.840 (0.75–0.92)	0.820	0.76	0.71
Hybrid 4-way (ConvNeXt + MP)	0.884 (0.81–0.95)	0.882	0.86	0.76
Hybrid best (VSD+Aorta+RVOT, ConvNeXt + MP)	0.890 (0.82–0.95)	0.888	0.83	0.74
Whole-heart baseline (ResNet18)	0.793 (0.69–0.88)	0.801	0.62	0.76
Radiomics (XGBoost) 4-way	0.731	—	—	—
CT-FM (frozen) 4-way	0.748	—	—	—

MP, multi-phase; CT-FM, CT foundation model. CIs from a 2000-sample patient-level bootstrap. No ablation (radiomics, CT-FM, 3D CNN, attention-MIL) exceeded the 2.5D hybrid. The ConvNeXt multi-phase rows are exploratory optimizations, not ablations; their four-component gain has a paired-bootstrap CI marginally excluding zero (DeLong *p* = 0.059). CIs are not available for the radiomics and CT-FM rows. All hybrids use mean fusion.

**Table 4:** Region-specific AUROC increments from the backbone × phase factorial (out-of-fold, n = 84). Baseline is the ImageNet-ResNet18 single-phase model; the backbone increment is ResNet18 → ConvNeXt-tiny with single-phase data; the multi-phase increment is the further change when all available cardiac phases are added on top of the ConvNeXt backbone. All increments lie within the bootstrap 95% CI of the corresponding AUROC (Supplementary Table S4). Each column is rounded independently, so a row may not sum exactly to its final value. Regions are named by intended target; see Table 5 for containment.

Region	Baseline	Backbone Δ	Multi-Phase Δ	Final
VSD (malalignment septal defect)	0.828	+0.029	−0.001	0.857
Over-riding aorta	0.786	+0.007	+0.071	0.863
RVOT	0.800	+0.016	+0.051	0.867
RVH	0.769	+0.013	+0.019	0.801

**Table 5:** Containment of the physician region-of-interest annotations within each fixed prior. “Centre in box” is the fraction of annotations whose centre lies inside the prior; “in sampled band” is the fraction whose centre lies inside the z range the model actually presents to that branch, which spans only the central 30–70% of the region depth. Both readers are pooled; per-reader centre-in-box values agree to within 4 percentage points for every region, and per-reader in-band values to within 11 percentage points. Distance is the median absolute displacement beyond the nearest band edge, over the annotations that fall outside it; the count of such annotations is given in parentheses. Every region has annotations outside the band, but only for the RVOT is the displacement large.

Region	Annot.	Sampled Band	Centre in Box	In Band	Median Displacement
Aorta	159	z 44–84	83.0%	84.3%	5.4 slices (25 of 159)
VSD	163	z 47–81	96.3%	58.9%	5.7 slices (67 of 163)
RVH	152	z 47–81	43.4%	53.9%	8.2 slices (70 of 152)
RVOT	162	z 29–59	48.1%	0.6%	21.6 slices (≈12.5 mm; 161 of 162)

**Table 6:** What the model uses, and whether correcting the priors helps. Occlusion importance is the mean fall in the predicted probability when a $24^{3}$ cube is removed, computed on the whole-heart baseline model (AUROC 0.793), which uses no regional prior, and averaged over the 42 TOF patients; the control is the mean importance of grid cells lying within 8 voxels of the ROI cell’s own radius from the volume centre, in the same patient. Occlusion *p*-values are from a 10,000-sample paired sign-flip permutation test over patients, and corrected-prior *p*-values from a 10,000-sample paired patient-level bootstrap. Ratios are computed from unrounded means, so they may differ in the last digit from the ratio of the rounded values printed here. Corrected priors keep the published box sizes but move the centre to the median physician annotation of the training controls only, recomputed within each fold, so no held-out patient and no TOF anatomy inform the box they are cropped with. As a further control, boxes of the same sizes were placed at random positions inside the standardized heart (five independent placements per size, each applied identically to every patient): mean AUROC 0.765 (SD 0.036, range 0.667–0.823), against 0.796 for the four published priors, 0.793 for the whole-heart baseline and 0.651–0.663 for morphometry alone.

	Occlusion Importance			Corrected Prior		
Region	At ROI	Control	Ratio (p)	Published	Corrected	Δ (p)
VSD	0.0547	0.0190	2.88 (0.0001)	0.828	0.797	−0.031 (0.46)
Aorta	0.0409	0.0226	1.81 (0.0098)	0.786	0.813	+0.027 (0.32)
RVH	0.0309	0.0075	4.13 (0.0012)	0.769	0.779	+0.010 (0.86)
RVOT	0.0197	0.0159	1.24 (0.50)	0.800	0.883	+0.083 (0.10)

**Table 7:** Deep-learning studies of tetralogy of Fallot and congenital heart disease, ordered by modality. The unit of analysis used for train–test splitting is the column that most strongly governs comparability: image- or slice-level splitting places near-duplicate images from the same patient on both sides of the split. Confounder control is reported as stated by the authors.

Study	Modality and Task	Patients	Split Unit	Confounder Control	Headline Metric
This study	Cardiac CT; TOF vs. control	84	Patient	1:1 age and sex matching; single reconstruction	AUROC 0.829 (0.74–0.91)
Wang et al. [10]	Cardiac CT; TOF vs. control	20 (80 slices)	Slice	None	AUC 0.96
Xu et al. [7]	Cardiac CT; 17-class CHD	>3750	Patient	None	Accuracy 86.0%
Xu et al. [8]	Cardiac CT; multi-class CHD	110	Patient	None	Accuracy 72.5% full prediction, 82.0% selective (88.4% coverage); TOF 7/12 correct
Yao et al. [9]	Cardiac CT; PA indices, surgical triage	122	Patient	None	Triage AUC > 0.87
Gao et al. [46]	Echocardiography; TOF diagnosis	1986	Patient	Explicit “TOF mimics” comparator class	AUROC 0.989 (0.977–0.992)
Yu et al. [47]	Foetal echo; TOF vs. VSD	Not reported	Image	None	AUC 0.873
Nurmaini et al. [48]	Foetal echo; septal-defect detection	50	Both reported	None	mAP 98.3% intra- vs. 82.4% inter-patient
Arnaout et al. [49]	Foetal ultrasound; CHD screening	1326/4108	Patient and study	Gestational-age window	AUC 0.99
Tilborghs et al. [6]	Cardiac MRI; repaired-TOF quantification	232 (132 TOF)	Patient	None	Dice 92.9% (RV)

## Data Availability

Owing to ethical and patient-privacy restrictions on paediatric cardiac imaging, the data are not publicly available. De-identified derived data are available from the corresponding author upon reasonable request, subject to approval by the Başakşehir Çam and Sakura City Hospital Scientific Research Ethics Committee No. 1. A confidential package can be provided to editors and reviewers during peer review. The analysis code (preprocessing, confounder matching, fold assignment, model training, evaluation, and statistical-analysis scripts) contains no patient data and can likewise be supplied as a confidential package to editors and reviewers on request.

## Supplementary Materials

The following supporting information can be downloaded at https://www.mdpi.com/article/10.3390/diagnostics16172814/s1, Figure S1. Per-fold AUROC across the five cross-validation folds; Table S1. Training hyperparameters; Table S2. Per-fold AUROC (single 5-fold split, 2.5D, out-of-fold); Table S3. Repeated cross-validation (optimism-corrected; 20 stratified 5-fold splits, 2.5D); Table S4. Backbone × multi-phase factorial (per-component AUROC, out-of-fold); Table S5. 3D baseline—full vs. light augmentation (per-component AUROC); Table S6. CT-FM foundation-model ablation (per-component and hybrid AUROC, out-of-fold); Tables S7. CLAIM checklist (42-item), completed item-by-item; Tables S8. TRIPOD + AI checklist (27-item), completed item-by-item; Table S9. Participant flow (STARD-style); Table S10. Permutation paired-AUROC sensitivity test (2.5D vs. 3D, per component); Table S11. Multi-phase phase-count distribution by group (multi-phase experiment); Table S12. Grad-CAM faithfulness: insertion/deletion against two null attributions; Table S13. Grad-CAM sanity checks (Adebayo et al.); and Table S14. Reported effect sizes for domain-specific pretraining (Discussion, Future Directions) (reported effect sizes for domain-specific pretraining).

## Abbreviations

The following abbreviations are used in this manuscript:

TOF	Tetralogy of Fallot
CT	Computed tomography
VSD	Ventricular septal defect
RVOT	Right-ventricular outflow tract
RVH	Right-ventricular hypertrophy
CNN	Convolutional neural network
AUROC	Area under the receiver-operating-characteristic curve
AUC	Area under the curve
AUPRC	Area under the precision–recall curve
ROI	Region of interest
CI	Confidence interval
Grad-CAM	Gradient-weighted Class Activation Mapping
CLAIM	Checklist for Artificial Intelligence in Medical Imaging
