# Supplementary material for: Confounder-Matched Deep Learning on Cardiac CT for the Diagnosis of Tetralogy of Fallot: A Proof of Concept
Source: Diagnostics (Basel). 2026 Sep 1;16(17):2814. doi: 10.3390/diagnostics16172814 (PMC13565130; doi:10.3390/diagnostics16172814)
Supplement: Supplementary file 1 [file diagnostics-16-02814-s001.zip › diagnostics-4432913-supplementary.pdf]

Supplementary Materials — Confounder-Matched Deep Learning on Cardiac CT for the Diagnosis of Tetralogy of Fallot: A Proof of Concept

Supplementary Materials

Elnur Karimov [1,2,†], İnci Zaim Gökbay [1,3,*] and Serap Baş [4] — for *Diagnostics* (MDPI).

[1] Institute of Graduate Studies in Health Sciences, İstanbul University; [2] Clinic of Pediatric Cardiology, Başakşehir Çam and Sakura City Hospital, University of Health Sciences Turkey; [3] Department of Artificial Intelligence and Data Engineering, İstanbul University; [4] Clinic of Radiology, Başakşehir Çam and Sakura City Hospital, University of Health Sciences Turkey. * Correspondence: inci.gokbay@istanbul.edu.tr. † Derived from the doctoral (PhD) thesis of E.K. ORCID: E.K. 0000-0003-3711-2922; İ.Z.G. 0000-0002-4488-1642; S.B. 0000-0002-3607-0620.

This supplement provides the participant-flow table, per-fold cross-validation results, full training hyperparameters, the optimization/baseline ablations, the permutation and multi-phase sensitivity analyses, and the CLAIM and TRIPOD+AI reporting-checklist mappings referenced in the main text.

> All tables (S1–S14), including the participant-flow (STARD) table, the repeated cross-validation, the backbone×phase factorial, the full-augmentation 3D baseline, the CT-FM native-HU/fine-tune ablation, the permutation paired-AUROC sensitivity test, and the multi-phase phase-count distribution, are reported below.

Figure S1. Per-fold AUROC across the five cross-validation folds

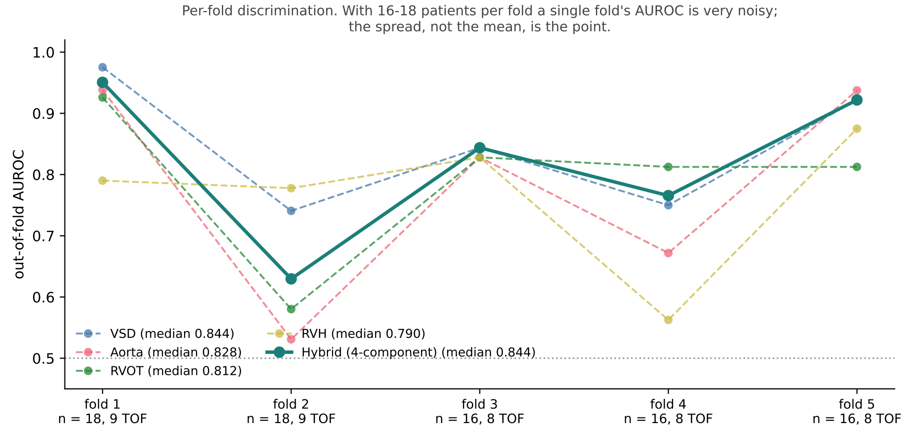

Per-fold AUROC for each tetrad component and the four-component hybrid (dots = folds; red line = median). Small folds (16–18 patients, 8–9 TOF) yield wide fold-to-fold variability, consistent with the bootstrap CIs in the main text.

Table S1. Training hyperparameters

Setting	2.5D transfer learning	3D CNN
Backbone	ImageNet ResNet18 (fc to 2 units); ConvNeXt-tiny / EfficientNet-B0 in optimization	MONAI DenseNet121 (spatial_dims=3)
Input	3-adjacent-slice axial slabs, 8 z-levels evenly spaced over central 30–70%, resized 3×112×112, ImageNet-normalized (~8 slabs/patient)	region resized to 64^3
Aggregation	unweighted mean of slab probabilities	—
Optimizer	Adam, lr 1e-4, weight decay 1e-4	Adam, lr 1e-3, weight decay 1e-4
Epochs	20	30 (40 for the full-augmentation variant)
Batch size	16	8
Loss	cross-entropy	cross-entropy
Augmentation	random axial flips + ±5% intensity scaling	flips + ±5% intensity (light); + rotation/scale/elastic (full)
Hardware / SW	Apple MPS GPU; PyTorch 2.9, MONAI 1.5, torchvision; fixed seeds	same

Fusion: per-component out-of-fold probabilities combined by (i) unweighted mean (pre-specified primary) and (ii) logistic-regression stacking. Evaluation: leak-free patient-level stratified 5-fold CV; 95% CIs from a 2000-sample patient-level bootstrap; AUROC comparisons by DeLong test with Benjamini–Hochberg correction (family = seven comparisons: the four within-component 2.5D-vs-3D contrasts, the two hybrid-vs-VSD contrasts, and hybrid vs. whole-heart). Grad-CAM: gradients into ResNet18 `layer4`.

Architectures of the comparator models (Reviewer 1). The 3D CNN is MONAI's `DenseNet121` with `spatial_dims=3`, `in_channels=1`, `out_channels=2`: an initial 7×7×7 stride-2 convolution and max-pool stem, followed by four dense blocks of 6, 12, 24 and 16 bottleneck layers (each a 1×1×1 → 3×3×3 BN-ReLU-Conv pair) with growth rate 32, separated by transition layers that halve channel count and spatial resolution, ending in global average pooling and a linear classifier (~11.2 M parameters). The CT foundation model is CT-FM's `SegResEncoder` (`project-lighter/ct_fm_feature_extractor`), a residual 3D encoder pretrained contrastively on ~148,000 CT volumes; input volumes were resampled to 96^3 and the deepest bottleneck stage was global-average-pooled to a feature vector. Three configurations were evaluated: frozen features on our [0,1] cardiac-windowed volumes; frozen features on natively preprocessed Hounsfield-unit volumes re-extracted with CT-FM's own intensity range ([−1024, 2048] → [0,1]); and fine-tuning, in which the deepest encoder stage (`layers[-1]`) plus a linear head were unfrozen while all earlier stages remained frozen.

Table S2. Per-fold AUROC (single 5-fold split, 2.5D, out-of-fold)

Component	Fold 1	Fold 2	Fold 3	Fold 4	Fold 5	Median (range)
VSD	0.975	0.741	0.844	0.750	0.922	0.844 (0.74–0.98)
Overriding aorta	0.938	0.531	0.828	0.672	0.938	0.828 (0.53–0.94)
RVOT	0.926	0.580	0.828	0.812	0.812	0.812 (0.58–0.93)
RVH	0.790	0.778	0.828	0.562	0.875	0.790 (0.56–0.88)
**Hybrid (4-way)**	0.951	0.630	0.844	0.766	0.922	**0.844 (0.63–0.95)**

Fold sizes: 18, 18, 16, 16, 16 patients (TOF per fold: 9, 9, 8, 8, 8).

Table S3. Repeated cross-validation (optimism-corrected; 20 stratified 5-fold splits, 2.5D)

Region	Mean AUROC	SD	Min	Max
VSD	0.807	0.030	0.736	0.844
Overriding aorta	0.772	0.027	0.725	0.817
RVOT	0.770	0.036	0.721	0.871
RVH	0.743	0.031	0.698	0.815
**Hybrid (4-way)**	**0.811**	**0.026**	**0.761**	**0.858**

The repeated-CV hybrid (0.811 ± 0.026) is ~0.018 below the single-split estimate (0.829), quantifying a modest single-split optimism. Nested cross-validation over the model-selection step is reported in Section 3.5 of the manuscript (AUROC 0.787 ± 0.029).

Table S4. Backbone × multi-phase factorial (per-component AUROC, out-of-fold)

Component	ResNet18 · single	ConvNeXt · single	ConvNeXt · multi	Backbone Δ (single-phase)	Multi-phase Δ (on ConvNeXt)
VSD	0.828	0.857	0.857	+0.029	−0.001
Overriding aorta	0.786	0.793	0.863	+0.007	+0.071
RVOT	0.800	0.816	0.867	+0.016	+0.051
RVH	0.769	0.782	0.801	+0.013	+0.019

The optimization gain is backbone-driven and region-specific: the stronger backbone adds a small amount everywhere, while multi-phase data helps substantially only for the aorta and RVOT. All increments fall within the ~±0.085 bootstrap CI.

Table S5. 3D baseline — full vs. light augmentation (per-component AUROC)

Component	3D · light aug	3D · full aug	2.5D (reference)
VSD	0.656	0.616	0.828
Overriding aorta	0.636	0.607	0.786
RVOT	0.573	0.659	0.800
RVH	0.596	0.573	0.769

Full augmentation (random rotation, scaling, elastic deformation + flips + intensity; 40 epochs) did **not** improve the 3D CNN over light augmentation, and the 3D CNN remained far below the 2.5D model. The 2.5D advantage is therefore robust to 3D regularization, not an artifact of an under-augmented baseline.

Table S6. CT-FM foundation-model ablation (per-component and hybrid AUROC, out-of-fold)

Configuration	VSD	Aorta	RVOT	RVH	Whole-heart	Hybrid (4-way)
Frozen, cardiac-window proxy	—	—	—	—	0.798	0.748
Frozen, native HU (−1024..2048)	0.622	0.674	0.741	0.777	0.797	0.779
Fine-tuned, native HU	0.724	0.720	0.693	0.741	—	0.769
**2.5D pipeline (reference)**	**0.828**	**0.786**	**0.800**	**0.769**	**0.793**	**0.829**

Native-HU preprocessing improved the frozen CT-FM hybrid (0.748 to 0.779), confirming that the original cardiac-window proxy mildly handicapped it; fine-tuning the encoder's deepest stage did not help further (0.769), consistent with over-fitting a 77M-parameter encoder on 84 patients. In every configuration CT-FM remained below the fine-tuned 2.5D hybrid; the frozen CT-FM whole-heart feature marginally exceeded the 2.5D whole-heart (0.798/0.797 vs. 0.793, within the bootstrap CI) but stayed well below the 2.5D hybrid.

Table S7. CLAIM checklist (42-item), completed item-by-item

Checklist for Artificial Intelligence in Medical Imaging (Mongan J, Moy L, Kahn CE. *Radiol. Artif. Intell.* 2020;2(2):e200029). Every item is mapped to the section, table, figure, or page where it is addressed; items the study does not address are marked ''Not reported''. Page numbers refer to the typeset proof (diagnostics-4432913, 31 pp.).

#	Item	Where addressed in the manuscript
1	Identify the study as applying AI/ML methodology and name the technology category (e.g., deep learning) in the title and/or abstract.	Title ("…Deep Learning on Cardiac CT…"); Abstract ("deep-learning pipeline," 2.5D transfer learning/ImageNet ResNet18, 3D CNN DenseNet121). Keyword list also lists deep learning, transfer learning. [p. 1]
2	Provide a structured summary of study design, methods, results, and conclusions.	Abstract — structured as Background/Objectives, Methods, Results, Conclusions; states retrospective single-scanner design, matched cohort (n=84), and headline AUROC results. [p. 1]
3	State the scientific/clinical background and the intended use and clinical role of the AI approach.	§1 (Introduction) — TOF epidemiology and the growing role of cardiac CT; state of AI-in-TOF research; frames the intended role as a methodologically sound benchmark rather than an autonomous diagnostic aid. [p. 2]

#	Item	Where addressed in the manuscript
4	Define the clinical/scientific question and state the hypothesis/objective a priori.	§1 (Introduction), final two paragraphs — objective to develop and internally evaluate a confounder-controlled, anatomy-guided pipeline; explicit methodological question of obtaining an honest internal estimate under confounder control at small scale; pre-specified contributions listed. [p. 2]
5	Indicate whether the study is retrospective or prospective.	§2.1 (Study Design and Ethics) — "retrospective, single-centre diagnostic-accuracy study"; also stated in Abstract. [pp. 1, 6]
6	Define the study goal (e.g., model creation, feasibility/proof of concept) and the algorithm's intended clinical role.	Title ("A Proof of Concept"); §2.1 (overriding design principle: control confounding); §4.2 and §5 (intended role — possible triage/pre-read flag or QC, augmenting not replacing expert reading; no standalone diagnostic indication claimed). [pp. 6, 22, 26]
7	State the source(s) of data and reference ethics review/consent.	§2.2 (single Canon Aquilion ONE scanner, named hospital, contrast-enhanced cardiac CT 2022–2026); ethics-committee approval and consent waiver in §2.1 and the Institutional Review Board / Informed Consent statements. [p. 6]
8	Define how, where, and when eligible participants/studies were identified, with inclusion/exclusion criteria.	§2.2 (TOF cohort vs structurally-normal control cohort; 207 source studies [123 control, 84 TOF]; exclusion of rendered-only and non-harmonizable studies); §2.4 (harmonization eligibility: FC15 kernel, 0.5 mm, $\geq$30 slices); Supplementary Table S9 (participant flow). [p. 6]
9	Describe data pre-processing steps (normalization, resampling, etc.) with software.	§2.5 (Preprocessing — HU loading, automatic heart localization via distance transform, crop and resample to 128^3 index grid, [-200,800] HU windowing, scale to [0,1]); software versions in §2.7 and Supplementary Table S1. [pp. 6, 8]

#	Item	Where addressed in the manuscript
10	Describe selection of data subsets and any manual/automated subsetting procedures, if applicable.	§2.4 (single-reconstruction restriction; when a patient had multiple qualifying series, the series with the most images was taken as the representative volume and the remainder used as the multi-phase set); §2.7 (multi-phase = all FC15/0.5 mm phases, 218 phase-volumes). [pp. 6, 8]
11	Define the predictor and outcome variables, mapping to Common Data Elements where applicable.	§2.2 (outcome: TOF vs structurally-normal control; predictor: the contrast-enhanced cardiac CT volume). No mapping to Common Data Elements is provided. [p. 6]
12	Describe how data were de-identified and the regulatory basis.	§2.3 (De-Identification — removal of DICOM PHI and private tags, patient re-coding, PatientIdentityRemoved=YES, header verification; burned-in identifiers checked by visual inspection; age/sex/study date deliberately retained for matching). [p. 6]
13	State clearly how missing data were handled.	§2.2 and §2.4 (incomplete/non-harmonizable studies were excluded rather than imputed: 7 rendered-only without a diagnostic volume, plus studies lacking a harmonizable series); Supplementary Table S9. No imputation was performed (imaging cohort). [p. 6]
14	Provide detailed definitions of the ground-truth/reference standard, sufficient for replication.	§2.2 (case/control label definition: preoperative native TOF vs children with structurally normal hearts). Echocardiography is noted as the established diagnostic reference standard for TOF (§1, §4.2). Physician ROI annotations used for the containment audit are defined in §2.6 and §2.9. Per-case diagnostic criteria used to assign the TOF label are not fully specified. [pp. 2, 6, 7, 9, 22]
15	Describe the rationale for choosing the reference standard and its potential errors/limitations.	Not explicitly reported for the diagnostic label. Echocardiography is identified as the established reference standard for TOF (§1, §4.2), but no explicit rationale or error/limitation statement for the label reference standard is given. [pp. 2, 22]

#	Item	Where addressed in the manuscript
16	Specify the number and qualifications of annotators and the annotation process.	§2.6, §2.9 and §3.8 (two independent physician readers annotated all four regions in all 84 patients; the reporting radiologist independently re-annotated RVH in the illustrative case). Qualifications are given only as physician / reporting radiologist; the source/adjudicators of the diagnostic (case-vs-control) label are not separately described. [pp. 7, 9, 17]
17	Specify the software used for manual/semi-automated/automated annotation, with version numbers.	Not reported — the annotation tool/software used for the physician region-of-interest annotations is not named (annotations described as axis-aligned ROIs).
18	Describe measurement of inter- and intra-rater variability and how discrepancies were resolved.	§3.8 (inter-reader agreement reported: per-reader centre-in-box within 4 percentage points for every region, in-band within ~10.5 pp; in Fig 2 the two readers' and radiologist's RVH placements agree to within 5.4–11.9 voxels). No formal kappa, no intra-rater test, and readers were pooled rather than reconciled by a discrepancy-resolution procedure. [p. 17]
19	Describe the intended sample size and how it was determined (e.g., power calculation).	§2.4 (convenience sample yielding 42 matched pairs, n=84) and §4.6 (achieved precision: bootstrap 95% CI half-width ≈0.085 at 42 events). No a priori power/sample-size calculation was performed. [pp. 6, 24]
20	Specify how data were assigned to training, validation (tuning), and testing partitions, with proportions.	§2.10 (leak-free patient-level stratified five-fold cross-validation, no separate external hold-out; folds of 16–18 patients); model-selection tuning nested inside an inner five-fold loop in §3.5; Supplementary Table S1. [pp. 9, 14]
21	Describe the level at which partitions are disjoint (image, study, patient, institution).	§2.10 (folds assigned by patient identifier before any slab or phase extraction, so every axial slab and every cardiac phase of a patient stays in the same fold — patient-level disjoint; multi-phase volumes also grouped by patient). [p. 9]

#	Item	Where addressed in the manuscript
22	Provide a complete, detailed description of the model structure (inputs, outputs, layers).	§2.7 (2.5D ImageNet ResNet18 on 3-adjacent-slice slabs; 3D MONAI DenseNet121; ConvNeXt-tiny/EfficientNet-B0; CT-FM configurations); full architecture specification (DenseNet121 blocks, CT-FM SegResEncoder, inputs/outputs) in Supplementary Table S1 and its architecture paragraph. [p. 8]
23	Specify names and version numbers of all software libraries, frameworks, and packages.	§2.7 (PyTorch 2.9, MONAI 1.5, torchvision; Adam optimizer; Apple-MPS GPU); Supplementary Table S1. [p. 8]
24	Indicate how model parameters were initialized (e.g., randomization, transfer learning source).	§2.7 (ImageNet-pretrained initialization for the 2.5D ResNet18 and the ConvNeXt/EfficientNet backbones; 3D DenseNet121 trained from scratch; CT-FM contrastively pretrained on ~148,000 CT volumes); transfer-learning rationale given in the same subsection. [p. 8]
25	Completely describe all training procedures and hyperparameters (augmentation, epochs, convergence).	§2.7 (learning rates, weight decay, batch sizes, epoch counts per model, cross-entropy loss, flip and ±5% intensity augmentation, no LR schedule/early stopping, final-epoch weights, fixed seeds); Supplementary Table S1. [p. 8]
26	Describe the method and performance parameters used to select the best-performing model.	§2.7 (no validation-based checkpoint selection — final-epoch weights taken in every case); §2.8 (pre-specified primary model = four-component mean hybrid, fixed before analysis); §3.5 (component subset and decision threshold selected inside an inner cross-validation loop for the nested analysis). [pp. 8, 9, 14]
27	For ensemble models, describe each constituent model and how outputs are weighted/combined.	§2.8 (Hybrid Fusion — per-component out-of-fold probabilities fused by unweighted mean [pre-specified primary] and by logistic-regression stacking over all subsets); §3.3 (stacking coefficients reported; mean fusion adopted as simpler/robust). [pp. 9, 11]
28	Describe the metric(s) used to measure model performance and relate to clinical requirements.	§2.10 (primary metric AUROC; also AUPRC, accuracy, sensitivity, specificity, calibration slope/intercept/Brier score, and decision-curve net benefit). [p. 9]

#	Item	Where addressed in the manuscript
29	Indicate the uncertainty of the performance metrics (e.g., standard deviation, confidence intervals).	§2.10 (2000-sample patient-level bootstrap 95% CIs; DeLong test for paired AUROC; Benjamini–Hochberg FDR over a seven-member family; 10,000-sample permutation and paired-bootstrap tests). Reported throughout §3 and Tables 2–3. [pp. 9, 10]
30	Analyze the robustness or sensitivity of the model to assumptions or initial conditions.	§3.4 (Optimization; backbone×phase factorial), §3.5 (nested cross-validation over model selection), §3.9 (Robustness/Ablations — radiomics/XGBoost, CT-FM in 3 configurations, 3D CNN light/full augmentation, attention-MIL); repeated cross-validation (§3.3); Supplementary Tables S3–S6. [pp. 11, 13, 14, 20]
31	Describe the methods used to explain or interpret the model's results.	§2.9 (Interpretability Analysis — Grad-CAM at ResNet18 layer4 and occlusion sensitivity on the whole-heart baseline; both validated: Grad-CAM tested against a resolution-matched random attribution and a centred-blob null plus Adebayo weight/label sanity checks); §3.7 (Feature Interpretation); §4.4; Supplementary Tables S12–S13. [pp. 9, 15, 23]
32	Describe validation or testing of the model on external data (or note the limitation if none).	Not done — no external or independent test set; evaluation is internal cross-validation only. The absence of external validation is explicitly acknowledged in §4.6 (Limitations), §4.7 (Future Directions), and §5 (Conclusions). [pp. 24, 25, 26]
33	Report the flow of participants/cases, using a diagram indicating inclusions and exclusions.	§3.1 (participant flow 207 → 148 acquisition-harmonized → 84 matched, with exclusion reasons and numbers dropped by matching); Supplementary Table S9 (STARD-style participant-flow table); Figure 1A (study-flow schematic). [p. 10]

#	Item	Where addressed in the manuscript
34	Report demographic and clinical characteristics of cases, including across partitions.	§3.1 and Table 1 (matched-cohort age [median/IQR, mean±SD, range] and sex for control vs TOF, with p-values); subgroup discrimination by sex and by age band in §3.6. Characteristics are given for the whole matched cohort; a per-fold demographic breakdown is not tabulated (single cohort with cross-validation). [pp. 10, 15]
35	Report the final model's performance on all data partitions (benchmarked appropriately).	§3.2–§3.3 (out-of-fold AUROC for each component, the pre-specified and best-subset hybrids, and the whole-heart baseline); Tables 2 and 3; per-fold values in Supplementary Table S2; optimism-corrected repeated-CV in §3.3 and Supplementary Table S3; nested-CV in §3.5. [pp. 11, 14]
36	Provide estimates of diagnostic accuracy and their precision (e.g., 95% CIs), with ROC/calibration analysis.	§3.2–§3.3 (AUROC with bootstrap 95% CIs, AUPRC, sensitivity, specificity); Figure 4 (ROC curves with CI bars); §3.6 and Figure 6 (calibration slope 0.67 [CI 0.41–1.16], Brier 0.169, reliability and decision curves). [pp. 11, 15]
37	Provide information to help understand incorrect results (e.g., confusion matrix, example misclassifications).	§3.6 and Figure 5 (representative real cases at the Youden operating point covering all four confusion-matrix quadrants, including one false negative [TOF called control, p=0.195] and one false positive [control called TOF, p=0.991]); operating-point sensitivity/specificity reported in §3.6. [p. 15]
38	Summarize limitations of the study, including potential bias, statistical uncertainty, and generalizability.	§4.6 (Limitations — single-centre, retrospective, n=84, wide CIs, no external validation, residual indication and tube-voltage confounders, RVH not diagnostically equivalent, region-audit and expert-annotation leakage caveats, compounded optimistic biases, matched-cohort generalizability); reinforced in §4.3–§4.5 and §5. [pp. 22, 24, 26]

#	Item	Where addressed in the manuscript
39	Describe the implications for practice, including intended use and/or clinical role and translation barriers.	§4.2 (Clinical Implications and the Nature of the Task — no standalone diagnostic indication; possible near-term use as automated QC or a triage pre-read flag; PPV/false-positive burden at true low prevalence; augment not replace expert reading); §5 (Conclusions — methodological baseline, external validation required before clinical use). [pp. 22, 26]
40	State the registration number and name of the registry (for applicable studies).	Not applicable — retrospective diagnostic-accuracy study, not a prospective clinical trial, so no trial registration. Institutional ethics-committee approval (decision no. 11, 15 Jan 2025) is reported in §2.1 and the Institutional Review Board statement. [p. 6]
41	State where the full study protocol / code can be accessed.	Data Availability Statement (analysis code — preprocessing, matching, fold assignment, training, evaluation, statistics — and de-identified derived data available from the corresponding author, and as a confidential package to editors/reviewers, on request); Methods (§2) and the Supplement document the protocol. No public protocol registry or code repository is provided. [p. 3]
42	Specify the sources of funding and other support and the role of funders.	Funding statement ("This research received no external funding"); Acknowledgments (derived from E.K.'s PhD thesis; discloses use of an LLM tool for language editing only); Conflicts of Interest statement ("no conflicts of interest").

Table S8. TRIPOD+AI checklist (27-item), completed item-by-item

TRIPOD+AI statement (Collins GS, Moons KGM, Dhiman P, et al. *BMJ* 2024;385:e078378); the 27 numbered items are shown with their official sub-items (a/b/c). Every item is mapped to where it is addressed; items not addressed are marked ''Not reported''. Page numbers refer to the typeset proof.

#	Item	Where addressed in the manuscript
1	Identify the study as developing/evaluating a multivariable prediction model, the target population, and the outcome to be predicted.	Title: "Confounder-Matched Deep Learning on Cardiac CT for the Diagnosis of Tetralogy of Fallot: A Proof of Concept" — names the method (deep learning), modality (cardiac CT), target/outcome (diagnosis of TOF).
2	Provide a structured abstract following the TRIPOD+AI for Abstracts checklist (objectives, data, methods, results, conclusions).	Abstract (structured: Background/Objectives, Methods, Results, Conclusions) — covers cohort, 1:1 matching, model, cross-validation, AUROC with 95% CI, calibration, and hypothesis-generating conclusion. [p. 1]
3a	Explain the healthcare context (diagnostic or prognostic) and rationale for developing/evaluating the model, with references to existing models.	§1 (Introduction), paragraphs 1–2 — TOF, role of cardiac CT, and prior AI/CHD models cited (Wang et al. slice-level TOF-CT; Xu et al. 17-class CHD; Yao et al. PA triage); explicitly frames the confounder-control gap. [p. 2]
3b	Describe the target population and intended purpose of the model in the care pathway, including intended users.	§1 (target = paediatric TOF-vs-control on CT; states the unmet need is a methodological benchmark, not an autonomous aid) and §4.2 (Clinical Implications: intended use as QC/pre-read triage augmenting expert radiologists, not autonomous diagnosis). [pp. 2, 22]
3c	Describe any known health inequalities between sociodemographic groups.	Not reported. Age and sex are treated as confounders to be matched (§2.4), but no known health inequalities between sociodemographic groups are described. [p. 6]
4	Specify the study objectives, including whether the study is development or validation (or both) of a prediction model.	§1 (final paragraph): "we develop a CT-based, confounder-controlled, anatomy-guided deep-learning pipeline for TOF and evaluate it internally"; also stated in Abstract (Background/Objectives). Development + internal evaluation only. [pp. 1, 2]
5a	Describe the sources of data separately for development and evaluation datasets, the rationale for using them, and representativeness.	§2.1 (retrospective, single-centre diagnostic-accuracy design) and §2.2 (single Canon Aquilion ONE scanner; TOF cases and structurally-normal controls; rationale and non-healthy nature of controls). Representativeness limits in §4.6. No separate/external evaluation dataset exists (internal cross-validation only). [pp. 6, 24]

#	Item	Where addressed in the manuscript
5b	Specify the dates of the collected participant data (start/end of accrual; end of follow-up if applicable).	§2.2: "Contrast-enhanced cardiac CT was acquired between 2022 and 2026." No follow-up (diagnostic, cross-sectional). [p. 6]
6a	Specify key elements of the study setting, including the number and location of centres.	§2.1 (single-centre) and §2.2 (Başakşehir Çam and Sakura City Hospital, University of Health Sciences Turkey, İstanbul; single scanner). One centre. [p. 6]
6b	Describe the eligibility criteria for study participants.	§2.2 (TOF cohort; controls referred for a suspected-but-excluded cardiovascular abnormality with structurally normal hearts; exclusion of 3D-only series and non-harmonizable series) and §2.4 (inclusion restricted to FC15 kernel, 0.5 mm, $\geq$30 slices). Flow in §3.1 and Supplementary Table S9. [pp. 6, 10]
6c	Give details of any treatments received and how they were handled during development/evaluation, if relevant.	§2.2: all TOF studies acquired preoperatively (native, unrepaired anatomy; no exposure to post-repair appearances); reiterated in §4.6 as a limitation. [pp. 6, 24]
7	Describe any data pre-processing and quality checking, including whether similar across relevant sociodemographic groups.	§2.3 (DICOM de-identification, verification), §2.5 (HU loading, automatic heart localization, 128ˆ3 resampling, [-200,800] HU windowing, [0,1] scaling), and §2.6 (automatic axial/coronal/sagittal QC montages per patient; no case excluded on region-quality grounds). Whether pre-processing behaved similarly across sociodemographic groups is not separately reported. [pp. 6, 7]
8a	Clearly define the predicted outcome and time horizon, how/when assessed, rationale, and consistency of assessment across sociodemographic groups.	§2.2 defines the binary outcome (TOF vs structurally-normal control). Diagnostic label; no time horizon. Reference standard = echocardiography (stated in §1 and §4.1/§4.2 as the diagnostic reference standard). Consistency of outcome ascertainment across sociodemographic groups not separately reported. [pp. 2, 6, 20, 22]

#	Item	Where addressed in the manuscript
8b	If outcome assessment requires subjective interpretation, describe the qualifications and demographic characteristics of the outcome assessors.	Not reported. TOF-vs-control labels derive from the established clinical (echocardiographic) diagnosis; qualifications/demographics of the diagnosing clinicians are not stated. (The two ROI readers and reporting radiologist in §2.6/§3.8 annotated regions for the containment audit, not the class label.) [pp. 7, 17]
8c	Report any actions to blind assessment of the outcome to be predicted.	Not reported. No blinding procedure is described for assignment of the TOF/control label (clinical ground truth). Note the related disclosure in §4.6 that physician ROI annotations were made with the diagnosis known — but that concerns the audit annotations, not the outcome label. [p. 24]
9a	Describe the choice of initial predictors (inputs) and any pre-selection before model building.	§2.6 (a priori choice of four fixed anatomical sub-volume priors — VSD, overriding aorta, RVOT, RVH — as the regional inputs; whole-heart baseline uses no decomposition) and §2.7 (radiomics comparator: 19 predefined intensity/GLCM features per region). Component-subset selection handled analytically (§2.8, §3.5). [pp. 7, 8, 9, 14]
9b	Clearly define all predictors, including how and when measured (and any blinding of predictor assessment).	§2.5 (image inputs in index space, windowing/scaling), §2.6 (exact voxel-index box definitions for each prior), and §2.7 (2.5D slab sampling across central 30–70%, resize to 3×112×112, ImageNet normalization; 3D 64^3 input; radiomics feature computation). Inputs derived automatically from the acquired CT (§2.2/§2.4). [pp. 6, 7, 8]

#	Item	Where addressed in the manuscript
9c	If predictor measurement requires subjective interpretation, describe the qualifications and demographic characteristics of the predictor assessors.	Not applicable for the model inputs, which are derived automatically (automatic heart localization and fixed geometric boxes, §2.5–2.6; no manual, structure-dependent slice selection, §2.7). The physician ROI annotators used only for the containment audit are described in §3.8 (two independent readers for all four regions in all 84 patients, plus the reporting radiologist's re-annotation of RVH) but are not model predictors; their formal qualifications/demographics are not tabulated. [pp. 6, 8, 17]
10	Explain how the study size was arrived at and justify that it was sufficient; include any sample-size calculation.	§2.4 (matching yielded 42 pairs / n=84 — a convenience/matched sample; no formal a priori sample-size calculation). Adequacy discussed in §4.6 (42 events; bootstrap 95% CI half-width ≈0.085 — adequate for above-chance discrimination, not for resolving small model differences). Supplementary Table S8 labels it "convenience; matched n=84." [pp. 6, 24]
11	Describe how missing data were handled and give reasons for omitting any data.	§2.2 (exclusions: 7 studies with only 3D-rendered series; studies lacking a harmonizable series), §2.4, and §3.1 with full exclusion reasons in Supplementary Table S9 (participant flow). No per-feature imputation applies (whole-image inputs). [pp. 6, 10]
12a	Describe how the data were used (development and evaluation of performance), including any data partitioning and sample-size considerations.	§2.10 (leak-free, patient-level, stratified five-fold cross-validation; folds assigned by patient ID before slab/phase extraction; 20-repeat repeated CV for optimism) and §3.5 (nested CV for the selection steps). No hold-out external set. [pp. 9, 14]
12b	Describe how predictors were handled in the analysis (functional form, rescaling, transformation, standardisation).	§2.5 (HU windowing to [-200,800], scaling to [0,1], independent-axis resampling to 128^3) and §2.7 (slab resize to 3×112×112 with ImageNet normalization; 3D region resampled to 64^3; radiomics at 16 grey levels). CT-FM native-HU re-extraction ([-1024,2048]→[0,1]) in §2.7 and Supplementary Table S1. [pp. 6, 8]

#	Item	Where addressed in the manuscript
12c	Specify the model type and rationale, all model-building steps including hyperparameter tuning, and the method for internal validation.	§2.7 (model types: 2.5D ImageNet-ResNet18, 3D MONAI-DenseNet121, ConvNeXt-tiny/EfficientNet-B0, attention-MIL, CT-FM, radiomics/XGBoost — with rationale for the 2.5D transfer-learning choice), §2.8 (hybrid fusion), §2.10 (internal validation scheme), and §3.5 (nested CV over component-subset and threshold selection). Full hyperparameters and fixed seeds in Supplementary Table S1. [pp. 8, 9, 14]
12d	Describe if/how heterogeneity in model parameters and performance was handled and quantified across clusters (e.g., hospitals, countries).	Not applicable — single centre, single scanner (§2.1–2.2), so there are no hospital/country clusters. (Cross-fold variability is instead reported in §3.3 and Supplementary Tables S2–S3.) [pp. 6, 11]
12e	Specify all measures and plots (and rationale) used to evaluate performance (discrimination, calibration, clinical utility) and to compare models.	§2.10 — AUROC (primary), AUPRC, accuracy, sensitivity, specificity, calibration (slope, intercept, Brier), decision-curve net benefit; DeLong test and permutation test for paired AUROC comparison; 2000-sample patient-level bootstrap CIs; Benjamini–Hochberg correction over a pre-declared seven-member family. [p. 9]
12f	Describe any model updating (e.g., recalibration) arising from evaluation, overall or by group/setting.	§2.10 and §3.6 (temperature-scaling recalibration pre-specified and applied). Also §2.6/§3.8 describe leak-free prior repositioning ("corrected priors" from training-control medians) as an input-side update evaluated within folds. [pp. 7, 9, 15, 17]
12g	For model evaluation, describe how model predictions were calculated (formula, code, object, API).	§2.7 (patient-level probability = unweighted mean of per-slab probabilities; hybrid = mean of component probabilities, §2.8). Analysis code (preprocessing, matching, fold assignment, training, evaluation) available as a confidential package per the Data Availability Statement; fixed seeds/splits in Supplementary Table S1. [pp. 8, 9]

#	Item	Where addressed in the manuscript
13	If class-imbalance methods were used, state why and how, and any subsequent recalibration.	No resampling/class-imbalance technique was used; classes were balanced by design via 1:1 age- and sex-matching (§2.4), giving an artificial 50% prevalence. The consequences are addressed rather than corrected: AUPRC is flagged as inflated by the 1:1 balance (§2.10), and net benefit/PPV are discussed at the true far-lower prevalence (§3.6, §4.2). [pp. 6, 9, 15, 22]
14	Describe any approaches used to address model fairness and their rationale.	No explicit fairness-mitigation approach is reported. The closest elements are confounder matching on age and sex (§2.4, for internal validity) and subgroup performance by sex and age band (§3.6). Fairness per se (equity across sociodemographic groups) is Not reported. [pp. 6, 15]
15	Specify the model output (probabilities, classification); give details/rationale for any classification and how thresholds were identified.	§2.7 (output = class probability; patient probability = mean of slab probabilities). Thresholds: §2.10 (fixed 0.5 default), §3.6 (Youden operating point 0.37, in-sample/optimistic), and §3.5 (nested-CV-selected thresholds ranging 0.17–0.82, median 0.42). [pp. 8, 9, 14, 15]
16	Identify any differences between development and evaluation data in setting, eligibility, outcome, and predictors.	Not applicable — there is no separate evaluation (external) dataset; all estimates come from internal cross-validation of one cohort. The absence of external validation is stated explicitly in §4.6 and §5. [pp. 24, 26]
17	Name the IRB/ethics committee that approved the study and describe informed consent or its waiver.	§2.1 (Başakşehir Çam and Sakura City Hospital Scientific Research Ethics Committee No. 1, decision no. 11, 15 Jan 2025; Declaration of Helsinki; written-consent requirement waived for retrospective de-identified imaging). Restated in the Institutional Review Board Statement and Informed Consent Statement (back matter). [p. 6]
18a	Give the source of funding and the role of the funders for the present study.	Funding statement (back matter): "This research received no external funding."
18b	Declare any conflicts of interest and financial disclosures for all authors.	Conflicts of Interest statement (back matter): "The authors declare no conflicts of interest."

#	Item	Where addressed in the manuscript
18c	Indicate where the study protocol can be accessed, or state that a protocol was not prepared.	Not reported. No statement on the availability of a study protocol is provided (the manuscript refers to pre-specified analysis choices but does not point to an accessible protocol document, and does not state that no protocol was prepared).
18d	Provide registration information (register name and number), or state that the study was not registered.	Not reported. The manuscript gives no study-registration information and does not explicitly state that the study was unregistered.
18e	Provide details of the availability of the study data.	Data Availability Statement (back matter): data not publicly available due to paediatric privacy restrictions; de-identified derived data available from the corresponding author on reasonable request subject to ethics approval; a confidential package can be provided to editors/reviewers.
18f	Provide details of the availability of the analytical code.	Data Availability Statement (back matter): analysis code (preprocessing, matching, fold assignment, model training, evaluation, statistics) contains no patient data and can be supplied as a confidential package to editors/reviewers on request. Fixed seeds and per-fold splits noted in §2.7 and Supplementary Table S1. (Not deposited in a public repository.) [p. 8]
19	Provide details of any patient and public involvement in design, conduct, reporting, interpretation, or dissemination, or state no involvement.	Not reported. No patient-and-public-involvement statement is present.
20a	Describe the flow of participants through the study, including numbers with/without the outcome (a diagram may help).	§3.1 (207 source studies → 148 acquisition-harmonized → 84 matched, with exclusion reasons), Figure 1 (study-flow diagram), and Supplementary Table S9 (STARD-style participant flow: 42 TOF / 42 control). [p. 10]

#	Item	Where addressed in the manuscript
20b	Report characteristics overall and by source/setting (key dates, predictors incl. demographics, treatments, sample size, outcome events, follow-up, missing data); report differences across key demographic groups.	Table 1 (matched-cohort age and sex by group, with p-values) and §3.1 (age/sex balance, tube-voltage residual imbalance, infant-predominant age band, dropped-vs-retained comparison); Figure 3 (age distribution before/after matching). Single source/setting, so no by-source breakdown. [p. 10]
20c	For model evaluation, show a comparison with the development data of the distribution of important predictors (demographics, predictors, outcome).	Not applicable — no separate evaluation dataset. Related distributional comparisons are still shown internally: before-vs-after-matching and dropped-vs-retained distributions in §3.1 and Figure 3, and matched-group balance in Table 1. [p. 10]
21	Specify the number of participants and outcome events in each analysis (development, tuning, evaluation).	§3.1 (42 TOF, 42 control; n=84), §3.3 (per-fold sizes 16–18 patients, 8–9 TOF each), and Supplementary Table S2 (fold sizes 18/18/16/16/16; TOF per fold 9/9/8/8/8). Multi-phase counts (218 phase-volumes) in §2.10/§3.4 and Supplementary Table S11. [pp. 9, 10, 11, 13]
22	Provide the full prediction model (formula, code, object, API) to allow predictions in new individuals and third-party evaluation, including access/reuse restrictions.	Architectures fully specified in Supplementary Table S1 (and the S1 narrative: DenseNet121 block structure, CT-FM SegResEncoder, ResNet18 head). Being a deep model, there is no closed-form equation; trained weights/code are available only as a confidential package on request (Data Availability Statement), so full open third-party reuse is restricted.
23a	Report model performance estimates with confidence intervals, including for key subgroups; consider plots.	§3.2–§3.6: per-component and hybrid AUROC with 95% CIs (Tables 2 and 3), AUPRC/sensitivity/specificity, calibration slope/Brier (§3.6), decision curve (Figure 6); ROC curves and bootstrap CIs (Figure 4). Subgroups by sex and age band in §3.6 (and per-branch age stratification in §4.6). [pp. 11, 15, 24]

#	Item	Where addressed in the manuscript
23b	If examined, report results of any heterogeneity in performance across clusters.	Not applicable — single centre, no clusters. Cross-fold heterogeneity is instead reported (per-fold AUROC range 0.63–0.95 for the hybrid, §3.3; Figure S1; Supplementary Tables S2–S3). [p. 11]
24	Report the results from any model updating, including the updated model and its subsequent performance.	§3.6 (recalibration by temperature scaling: slope 0.67→0.89 but ECE 0.097→0.129 and Brier 0.169→0.170 — no net reliability gain). §3.8 and Table 6 report the leak-free prior-repositioning update ("corrected priors"), which did not change discrimination (all p≥0.10). [pp. 15, 17]
25	Give an overall interpretation of the main results, including fairness, in the context of the objectives and previous studies.	§4 opening paragraphs (overall interpretation vs the confounder-control objective) and §4.1 (Comparison with the Literature, incl. Table 7). Subgroup/age discussion in §3.6 and §4.6; note fairness/equity is not framed explicitly (see item 14). [pp. 15, 20, 24]
26	Discuss study limitations (non-representative sample, sample size, overfitting, missing data) and their effects on bias, statistical uncertainty, and generalisability.	§4.6 (Limitations) — single-centre/retrospective/n=84, wide CIs, infant-only matched cohort, unmatched scan indication, native (pre-repair) anatomy only, region-containment failures, in-sample optimism of operating point/recalibration/subset selection, and absence of external validation; also cross-referenced optimism analyses in §3.5. [pp. 14, 24]
27a	Describe how poor-quality or unavailable input data should be assessed and handled when implementing the model.	Partially addressed: §2.5 (automatic heart-localization with a central-crop fallback if no heart-sized component is found — not triggered here) and §2.6 (automatic per-patient QC montages; no case excluded on region-quality grounds). Explicit deployment-time handling of poor-quality/unavailable inputs is otherwise Not reported (proof-of-concept, not deployment-ready). [pp. 6, 7]

#	Item	Where addressed in the manuscript
27b	Specify whether users must interact in handling the input data or use of the model, and the level of user expertise required.	§4.2 (Clinical Implications): envisaged near-term use is automated quality control or a pre-read triage flag augmenting — not replacing — expert paediatric-cardiac radiological interpretation; the pipeline is automatic, and any deployed threshold/net-benefit claim must be re-established at realistic prevalence on external data. [p. 22]
27c	Discuss next steps for future research, with a view to applicability and generalisability of the model.	§4.7 (Future Directions: external multi-centre prospective validation; unmatched-cohort propensity/IPW analysis; blinded voxel-level segmentation; paediatric-adapted in-domain pretraining; surgical-planning phenotyping) and §5 (Conclusions: external, multi-centre, prospective validation is the essential next step before any clinical use). [pp. 25, 26]

Table S9. Participant flow (STARD-style)

Stage	TOF	Control	Total
Source studies assessed for eligibility	84	123	207
Excluded — 3D-rendered series only, no diagnostic volume	—	—	7
Excluded — no harmonizable series (FC15 kernel, 0.5 mm, $\geq$30 slices)	—	—	52
Acquisition-harmonized cohort	79	69	148
Excluded by 1:1 age/sex optimal (Hungarian) matching	37	27	64
**Final matched analysis cohort**	**42**	**42**	**84**

The 59 studies removed before harmonization (7 + 52) comprised 5 TOF and 54 controls; restricting to a single reconstruction disproportionately removed controls (more heterogeneous acquisition). Matching then dropped 37 TOF and 27 controls; the dropped controls were markedly older than those retained (median 6.0 vs. 0.33 yr; $p = 1 \times 10^{-11}$), whereas dropped and retained TOF were of similar age (0.42 vs. 0.33 yr; $p = 0.31$). The matched cohort is therefore an infant/young population (Section 4.6).

Table S10. Permutation paired-AUROC sensitivity test (2.5D vs 3D, per component)

Component	ΔAUROC (2.5D − 3D)	DeLong p	BH q	Permutation p (10,000 paired patient-level swaps of which model owns which probability)
VSD	+0.172	0.010	0.034	0.011
Overriding aorta	+0.150	0.016	0.037	0.016
RVOT	+0.227	0.007	0.034	0.007
RVH	+0.173	0.037	0.065	0.037

The permutation test makes no large-sample (asymptotic-normal) assumption about the AUROC sampling distribution. Its p-values (0.007–0.037) closely track the DeLong p-values (0.007–0.037), so the 2.5D-over-3D advantage does not depend on the DeLong approximation; as with DeLong, the weakest comparison (RVH) is borderline after Benjamini–Hochberg correction.

Table S11. Multi-phase phase-count distribution by group (multi-phase experiment)

Group	Patients	Phase-volumes	Phases/patient: median (range)
TOF	42	119	3 (1–6)
Control	42	99	2 (1–10)
**Total**	**84**	**218**	—

The two groups differed in the number of cardiac phases available per patient (Mann–Whitney p = 0.023). The exploratory multi-phase variant (Table S4) therefore carries a residual phase-count imbalance between groups, an additional reason to treat its gains cautiously; the pre-specified single-phase primary analysis is unaffected.

Tables S7 and S8 are the completed, item-by-item CLAIM (42-item) and TRIPOD+AI (27-item) reporting checklists, mapping every item to the section, table, figure, or page of the manuscript where it is addressed; items the study does not address are marked "Not reported".

Table S12. Grad-CAM faithfulness: insertion/deletion against two null attributions

Perturbation on a 14×14 grid of 8×8-pixel cells (the Grad-CAM map is natively 4×4 at ResNet18 `layer4` for a 112×112 slab). Cells ranked by mean attribution; masking applied in pre-normalization windowed-intensity space, identically across the three adjacent-slice channels; all eight slabs of a patient perturbed to the same fraction and the eight probabilities re-averaged as in deployment. Deletion baseline: constant per-slab mean (**lower AUC is better**). Insertion baseline: Gaussian-blurred canvas, sigma = 8 px (**higher AUC is better**). Controls: `random` = 4×4 uniform noise upsampled identically to Grad-CAM, averaged over 10 independent draws; `centre` = centred Gaussian blob whose width matches the observed second spatial moment of the maps (mean sigma 41.3 px). Delta = Grad-CAM minus control, per patient; 95% CI from a 2000-sample patient-level bootstrap; q from Benjamini-Hochberg over the eight region-by-direction comparisons within each control family. n = 84 patients per row.

Region	Direction	Control	Grad-CAM AUC	Control AUC	Delta	95% CI	q
VSD	Deletion	random	0.314	0.344	-0.029	-0.050 to -0.007	0.025
VSD	Deletion	centre	0.314	0.357	-0.043	-0.076 to -0.012	0.072
VSD	Insertion	random	0.278	0.313	-0.036	-0.056 to -0.015	0.001

Region	Direction	Control	Grad-CAM AUC	Control AUC	Delta	95% CI	q
VSD	Insertion	centre	0.278	0.203	+0.075	+0.052 to +0.098	0.000
Aorta	Deletion	random	0.390	0.361	+0.029	+0.012 to +0.047	0.009
Aorta	Deletion	centre	0.390	0.423	-0.033	-0.056 to -0.010	0.009
Aorta	Insertion	random	0.385	0.378	+0.006	-0.011 to +0.023	0.459
Aorta	Insertion	centre	0.385	0.304	+0.081	+0.056 to +0.107	0.000
RVOT	Deletion	random	0.202	0.205	-0.003	-0.019 to +0.013	0.363
RVOT	Deletion	centre	0.202	0.254	-0.052	-0.074 to -0.031	0.000
RVOT	Insertion	random	0.213	0.209	+0.005	-0.014 to +0.024	0.688
RVOT	Insertion	centre	0.213	0.160	+0.053	+0.033 to +0.074	0.001
RVH	Deletion	random	0.257	0.288	-0.032	-0.052 to -0.011	0.024
RVH	Deletion	centre	0.257	0.333	-0.077	-0.103 to -0.050	0.000
RVH	Insertion	random	0.240	0.221	+0.019	+0.003 to +0.035	0.290
RVH	Insertion	centre	0.240	0.157	+0.084	+0.061 to +0.106	0.000

Reading. Against the centred blob, Grad-CAM is superior in all eight comparisons and significantly so in seven, so the maps carry information beyond the centre prior that heart-localization and HU windowing impose. Against the resolution-matched random attribution there is no consistent advantage: significantly better in two comparisons, significantly worse in two, indistinguishable in four. For the VSD region the two removal directions disagree (deletion favours Grad-CAM, insertion favours the null), a contradiction documented for fixed-value perturbation. No localization claim is therefore made.

Limitations of this evaluation, stated explicitly. (i) Masking creates out-of-distribution inputs, so part of any probability drop reflects distribution shift rather than information removal. (ii) Fixed-value imputation can leak class information through mask geometry. (iii) Results are baseline-dependent; the interpretable quantity is the ranking against a control, not the absolute AUC. (iv) Saliency-evaluation metrics are themselves of unproven reliability. (v) Because the input stacks three adjacent slices in the channel dimension and Grad-CAM sums over feature channels, the map carries no through-plane attribution. (vi) ResNet18 is a global-average-pooling architecture and the map is computed at the last convolutional layer, so this Grad-CAM is mathematically identical to CAM and to HiResCAM at that layer; no CAM variant would alter these numbers. (vii) The analysis is post-hoc and exploratory, added in revision, on the same single-centre cohort used for model development.

Table S13. Grad-CAM sanity checks (Adebayo et al.)

Spearman rank correlation between each perturbed Grad-CAM map and the corresponding trained-model map, computed on the **native 4×4 grid** (16 values) rather than the upsampled map, because rank correlation over 12,544 bilinearly interpolated pixels is inflated by the interpolation rather than by agreement. SSIM is reported on the upsampled map for comparability with the literature but is known to be the less discriminating of the two for this method. Five random seeds per stage, all four components, all five folds. The random-vs-random floor makes every other row interpretable.

Test	Spearman (mean)	SD	SSIM	n
**Floor: random vs. random**	+0.010	0.027	0.343	20
Cascading randomization (cumulative, top-down)				
… through `fc`	+0.137	0.086	0.207	100
… through `layer4`	-0.024	0.059	0.079	100
… through `layer3`	-0.073	0.098	0.078	100
… through `layer2`	-0.047	0.113	0.078	100
… through `layer1`	-0.134	0.108	0.077	100
… through `conv1`	+0.003	0.194	0.074	100
Independent randomization (one block at a time)				
`fc` only	+0.137	0.086	0.207	100
`layer4` only	-0.039	0.090	0.095	100
`layer3` only	+0.169	0.193	0.371	100
`layer2` only	+0.200	0.131	0.406	100
`layer1` only	+0.238	0.115	0.473	100
`conv1` only	+0.266	0.126	0.473	100
**Label randomization** (retrained, 3 permutations)	+0.208	0.068	0.407	60

Reading. Under cumulative top-down randomization the map collapses to the random-vs-random floor as soon as `layer4` is randomized and stays there, so the explanation is sensitive to the model weights it depends on. Under label randomization – labels permuted within the training folds and each branch retrained – Spearman falls from 1.0 to 0.21, so the explanation is also sensitive to the data. Grad-CAM therefore passes both checks on this cohort, consistent with Adebayo et al., who report that Grad-CAM (unlike Guided Backpropagation and Guided Grad-CAM) passes them; the widely cited "edge detector" result applies to the guided variants, not to Grad-CAM itself.

Expected structure, stated so it is not mistaken for a failure. Grad-CAM computed at `layer4` is a function of the `layer4` activations and the classifier weights only. Randomizing `layer3` and below *independently* therefore leaves a partially correlated map (Spearman 0.17-0.27, still far below 1.0 but above the 0.01 floor), because those blocks influence the map only through the forward pass. This is a structural property of the method at this depth, not evidence that the explanation ignores the model. Adebayo et al. state the same: Grad-CAM is sensitive to model weights when randomization is downstream of the last convolutional layer.

Caveat. Passing the model-parameter randomization test is weaker evidence than it appears: the result is metric-dependent, and under SSIM alone Grad-CAM clusters with methods that fail on rank-based metrics. We therefore report both metrics and treat the sanity checks as a necessary, not a sufficient, condition, alongside the faithfulness evaluation in Table S12.

Table S14. Reported effect sizes for domain-specific pretraining (Discussion, Future Directions)

Finding	Effect	Source
RadImageNet vs. ImageNet, small cohorts	up to +9.4% AUC (thyroid US 0.85 vs 0.76, P<.001)	Mei et al. 2022

Finding	Effect	Source
RadImageNet vs. ImageNet, largest cohort tested	+0.9% AUC	Mei et al. 2022
RadImageNet vs. ImageNet, panoramic radiographs	**-6.5% AUC** (95% CI -9.5 to -3.5, P<.01)	Okazaki et al. 2025
ImageNet transfer benefit attributable to semantic transfer	little; driven by over-parameterization and low-level feature reuse	Raghu et al. 2019
Adult-trained CT organ segmentation applied to children	mean Dice 0.73 vs 0.81 (P<.001); <0.30 for small structures in 0-4 y	Chatterjee et al. 2025
Volumetric self-supervised pretraining vs. from scratch	outperforms; annotation cost reduced >=30%	Zhou et al. 2021

No pretraining corpus or foundation model built for pediatric cardiac CT or congenital heart disease was identified; all CT foundation models surveyed are pretrained on predominantly adult data.
